# Divergence between predicted and actual perception of climate information

**DOI:** 10.1093/pnasnexus/pgaf084

**Published:** 2025-03-18

**Authors:** Amir Tohidi, Stefano Balietti, Samuel Fraiberger, Anca Balietti

**Affiliations:** Department of Information and Computer Science, University of Pennsylvania, 3317 Chestnut St, Philadelphia, PA 19104, USA; Chair of Data Science in the Economic and Social Sciences, Mannheim University, Schloss, Mannheim BW 68161, Germany; Development Impact Evaluation, The World Bank, 1818 H Street NW, Washington, DC 20433, USA; Alfred Weber Institute for Economics, Heidelberg University, Bergheimer Straße 58, Heidelberg BW 69115, Germany

**Keywords:** climate change, news articles, opinion shift, predictions, persuasion

## Abstract

Despite strong scientific consensus on the severe risks posed by climate change, a substantial segment of the population remains unconvinced, limiting progress on effective climate action. Persuading climate skeptics is essential for building broader support for stronger climate policies and accelerating efforts to mitigate climate change. However, outreach efforts often depend on perceptions of skeptics’ openness to climate communication: when persuasion is seen as unlikely, communication efforts tend to diminish. In this paper, we investigate the predicted versus actual impact of climate change information on skeptics. Using a series of surveys with US respondents, we first gather predictions about the effectiveness of authentic news articles in changing skeptics’ views. Our findings reveal a widespread pessimism: climate advocates expect no change in attitudes, while skeptics anticipate a backfire effect that reinforces their skepticism. Contrary to these predictions, our preregistered survey experiment finds that exposure to climate change articles significantly increases concern among skeptics. However, their responses vary in terms of willingness to adopt climate-friendly behaviors and support climate policies. These results reveal a significant disconnect between people’s expectations and actual effects of climate communication on skeptics, emphasizing the need for sustained and strategic investments in climate communication to foster greater public engagement and support for climate action.

**Significance Statement**

Despite a strong scientific consensus on the severe impacts of climate change, many individuals remain unconvinced, limiting effective policy responses. Persuading climate skeptics is essential for fostering greater support for climate action. However, efforts to communicate with skeptics often depend on pessimistic expectations about their responsiveness. Our research reveals that these expectations may be misplaced: exposure to factual climate change news increased concern among skeptics, challenging the belief that they are unmovable. These findings highlight the critical importance of sustained climate communication efforts in building broader consensus and driving more ambitious climate action.

## Introduction

The debate surrounding climate change policies has emerged as one of the most contentious issues in American politics (1–5). Despite mounting scientific evidence (6), the depth and pervasiveness of this rift have grown stronger over the years, leading to more pronounced partisan differences than on any other issue (7–10). Partisan media have played an active role in shaping polarization (11), facilitated by a tendency to consume homogeneous and proattitudinal news (12, 13), a pattern even stronger amongst political groups aligned with climate skepticism (14–16).

Despite a highly polarized environment, bipartisan cooperation remains critical to achieve effective climate action. In fact, the American political system is strongly influenced by party median voters (17–19), and therefore representatives might turn a deaf ear to climate calls at odds with the wishes of their copartisans. Furthermore, as known from the behavioral literature on collective action (20) and conditional cooperation (21–23), expectations of others’ behaviors significantly influence our own actions. If skeptics are perceived unresponsive to climate initiatives and unlikely to take climate action themselves, this perception can diminish engagement efforts and undermine cooperation. Experimental evidence highlight that communicating sentiment and outlook can lead to more positive cooperation, even within culturally diverse groups, in scenarios involving collective risks to society, such as climate change (24). However, if conflict is anticipated, individuals will avoid cross-cutting political communication altogether (25, 26), thereby obstructing collective climate action.

**Fig. 1.**
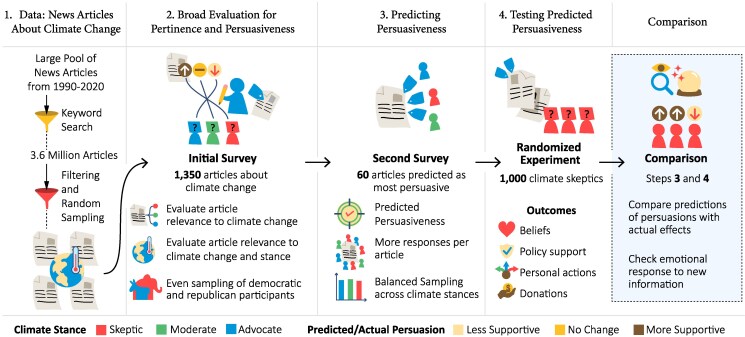
Experimental procedures. The study consists of four sequential steps. Step 1 involves the collection of a large number of news articles about climate change. Step 2 is about labeling a random sample of news articles for topical pertinence and persuasiveness on readers with different climate stance. Step 3—a refinement of step 2—creates the predictions that are tested in the preregistered experiment in step 4.

In this work, we use an iterative crowdsourced approach combined with a preregistered survey experiment to study the relationship between the expected and the actual persuasiveness of information about climate change on climate skeptics. We first ask survey respondents with different climate stances—namely advocates, moderates, and skeptics—to assess the persuasiveness of news articles selected from a large corpus of news about climate change. Then, in a preregistered experiment, we test the accuracy of these forecasts on various outcomes including climate change beliefs, support for climate policies, willingness to adopt climate-friendly behavior, and real-stake climate donations (see Fig. 1).

Understanding the relationship between expected and actual persuasiveness of information requires exploring the cognitive processes that shape how individuals predict others’ reactions. At the core of this is the concept of theory of mind, which refers to the ability to infer the thoughts, beliefs, and emotions of others (27–29). This cognitive skill plays a central role in forecasting the effects of persuasive messages, as it enables individuals to consider how others process and respond to information. In the context of climate change, the accuracy of such predictions may depend on a forecaster’s ability to empathize with or accurately model the mental states of individuals who share, or differ from, their own climate stance.

This variability in predictive ability may be shaped by psychological processes tied to group identity and shared perspectives. For example, social identity theory (30, 31) suggests that individuals are better at understanding and empathizing with members of their ingroup due to shared norms, values, and experiences. In the context of climate skepticism, this shared identity may allow skeptics to form deeper connections and anticipate how like-minded individuals react to persuasive messaging. Similarly, ingroup empathy (32, 33) posits that people are more attuned to the emotions, motivations, and thought patterns of those within their own group, further enhancing their ability to predict responses. Finally, projection bias (34, 35)—the cognitive tendency to assume that others share one’s own beliefs, attitudes, or reactions—may enable skeptics to project their own responses to climate change information onto others who share their worldview, thereby increasing predictive accuracy within their ingroup. Building on these theoretical considerations, we test the following hypothesis:

*H1*: Climate skeptics form more accurate predictions than other groups about the persuadibility of like-minded skeptics.

In the context of climate change, the task of persuasion presents significant challenges, given the apparent stability of beliefs (36). Individuals often resist changing deeply held beliefs when confronted with information that contradicts their worldview (37, 38). This resistance to belief change is frequently accompanied by heightened negative emotions due to the conflict between the perceived importance of their existing beliefs and the uncertainty introduced by the new information (39, 40). Neurological studies have demonstrated that challenging individuals’ political beliefs with counterevidence leads to increased activity in brain regions associated with self-representation and disengagement from the external world (41). A similar phenomenon could occur regarding climate change, where emotions are major drivers of climate perceptions and actions (42). Hence, in addition to *H1*, we test:

*H2*: Climate skeptics experience negative emotions in reaction to the articles supporting climate change.

The results of our study highlight a striking divergence between anticipated and actual shifts in opinions. While all climate stance groups express pessimism about the persuasive power of news articles—with climate skeptics even predicting a backfire effect—the randomized experiment reveals a significant positive impact of climate information on skeptics’ beliefs about climate change. The observed effect size exceeds 0.2 SDs (P<0.001) and, when contextualized against similar persuasion experiments (43), demonstrates not only statistical significance but also a substantial magnitude.

Despite the observed change in beliefs, support for climate policy, intended climate-friendly behavior, and real-stake donations remain unaffected by the treatment on aggregate. However, we find evidence of heterogeneity in treatment effects, with policy support increasing significantly among weaker skeptics, suggesting that preexisting attitudes play a critical role in moderating the treatment’s impact. These findings underscore a broader attitude–behavior gap, where shifts in beliefs do not necessarily translate into concrete actions or behavioral intentions. This gap has been documented across diverse contexts, including climate change (43–45), political attitudes (46, 47), immigration (48, 49), antidiscrimination policies (50), and preferences for redistribution (51, 52).

Overall, we find no evidence supporting *H1*, which posits that climate skeptics are better at predicting the persuadability of like-minded individuals. Instead, shared skepticism appears to hinder accurate predictions, challenging theories such as social identity theory and ingroup empathy, which argue that shared group membership enhances understanding. In highly polarized contexts like climate change, these theories may break down, as misperceived ingroup norms distort perceptions. This aligns with the perspective of You and Lee (53), who argue that individuals often perceive their group’s norms as more extreme than their own due to a desire to maintain ingroup distinctiveness and their tendency to base perceptions on highly visible prototypical members, such as outspoken public figures, whose views are more extreme than the group average. Applied to climate skepticism, these dynamics may lead skeptics to exaggerate the resistance of like-minded individuals to persuasion, contributing to their mispredictions. These findings underscore the need for a more nuanced understanding of prediction processes in polarized settings.

Furthermore, we find no evidence of a strong emotional response among climate skeptics exposed to climate change information. In the aggregate sample, neither positive nor negative emotions were significantly impacted (no support for *H2*), which may have facilitated belief updating and increased climate concerns. However, strong skeptics exhibited mild negative emotional reactions, suggesting that negative emotions are more pronounced among those with deeply entrenched skepticism and may limit belief change in such cases.

The primary aim of this study is to examine the divergence between predicted outcomes and actual effects. Given that the most significant discrepancies in predictions concerned the expected responses of skeptic readers, we focused our analysis on this group. While a study aimed at maximizing persuasion might instead target moderates, where consensus-building could be more efficient, such an approach falls outside the scope of our research. By focusing on skeptics, we sought to understand the most challenging cases, reasoning that if an intervention proves effective in this context, it should have good chances to succeed with more moderate audiences as well. Indeed, our heterogeneity analysis supports this hypothesis, as we observe that persuasion is stronger among “weak skeptics.”

Understanding the divergence between anticipated and real shifts in opinions provides valuable insights into the complexities of climate change communication and the effectiveness of public engagement strategies (54). This underscores the importance of leveraging complexity science to better anticipate and manage the intricate dynamics at play in public opinion and collective action, particularly by accounting for feedback loops and cascade effects that conventional strategies often overlook (55). Our results contradict prevailing assumptions about the fixed nature of climate skepticism and encourage an inclusive dialogue on climate change. Skeptics could display less opposition than anticipated, and polarization could be partially mitigated through careful and proper selection of persuasive messages.

## Materials and methods

The experimental procedures in this paper consist of four sequential steps: (1) retrieval of a large database of everyday news articles about climate change, (2) broad filtering of articles for pertinence and persuasiveness, (3) collecting prediction data about their persuasiveness, and (4) testing their actual persuasiveness. Steps (2)–(4) involved crowdsourced tasks conducted on Prolific and implemented in NodeGame (56). Table S1 presents the summary statistics of respondent characteristics for all surveys. The experiment was approved by the Institutional Review Board (IRB) at Massachusetts Institute of Technology (Exemption evaluation code: E-5038) and preregistered at AsPredicted.org.^a^ Prior to participating, all individuals were provided with an informed consent document. Participants were required to be 18 years of age or older to participate. The consent form outlined that mandatory questions were clearly marked and responses that appear to be random or negligent will be rejected. Participants were informed that there were no anticipated risks associated with the study, and that they could exit the study at any time without penalty if they experienced discomfort. Below, we outline key aspects of each design step, also illustrated in Fig. 1; additional details are provided in Supplementary Material section, Research design and crowdsourced tasks.

###  

#### Step 1: retrieval of a large database of everyday news articles about climate change

As a first step, we downloaded a large collection of 3.6 million English-language news articles containing the keywords “climate change” or “global warming” from the Factiva news aggregator.^b^ The dataset included articles published from 1990 to 2020, and we deliberately refrained from applying any preliminary filtering based on publication year, fully allowing respondents to decide which articles could potentially be more persuasive. Due to the large sample size, we first took a random sample of 36,000 articles. Then, to further ensure relevance, the articles were screened to include the keywords “climate change” or “global warming” either in the title or in the initial three sentences (57). Additionally, we limited our sample to articles up to 500 words to align with the content typically shared on social platforms, known for character constraints. This criterion also considered that individuals are less inclined to engage with lengthy content, particularly if it challenges their existing beliefs.

This procedure resulted in a collection of 4,819 news articles. To manage labeling costs effectively, we subsequently randomly selected a sample of 1,350 articles for analysis regarding predicted persuasiveness, which we will elaborate on in the following section. Summary statistic about source and year of publication can be found in Figs. S4, S5, and Table S2.

#### Step 2: evaluation for pertinence and persuasiveness

In a second step, using an online survey on Prolific with 798 participants, we evaluated the relevance and persuasiveness of each article regarding climate change. Four respondents, two democrats and two republicans, evaluated each article to ensure a balanced perspective across political affiliations. To ensure article relevance, respondents classified articles based on the extent to which their content addressed the topic of climate change. To evaluate persuasiveness, each respondent assessed how three hypothetical readers—supportive, indifferent, and opposing climate actions—might alter their beliefs about climate change after reading the article. We asked respondents to evaluate the persuasiveness for the readers across climate stances for two reasons. First, climate skeptics might feel unfairly targeted and therefore purposefully provide untrue estimates, if they are asked to evaluate the persuasiveness for skeptics only. Second, being confronted with readers with different climate stance compels respondents to think more critically and adapt their judgments to each stance. In total, 6,893 labels were collected, and we identified 550 articles where the majority of evaluators agreed that climate change was the prevailing theme. Further details about survey responses are presented in the Supplementary Material section, Research design and crowdsourced tasks, Figs. S2, S3, and S6.

#### Step 3: predicting persuasiveness

In the third step, we created a ranking of the most persuasive articles according to the climate stance of the labelers (skeptic, moderate, and advocate). Details about climate stance measurement is presented in Supplementary Material section, The climate stance index. For each stance group, we then selected about 20 articles that were predicted to be most persuasive for an hypothetical audience who does not believe in climate change and is against climate actions. This resulted in a final set of 60 climate-relevant articles predicted to be the most persuasive. These articles covered the topic of climate change comprehensively, employing diverse styles such as factual and statistical presentations, moral arguments, and personal stories. They addressed a variety of common themes, including the urgency of action, local and regional impacts, and economic consequences of climate change. A qualitative thematic analysis of the content of these 60 articles is presented in Table S3.

We then run a new online survey with 795 Prolific participants. Here, each of the selected 60 articles was reviewed for persuasiveness by a total of 30 participants balanced across their climate change stance (skeptic, moderate, and advocate). The distribution of labels across articles and stance groups can be seen in Fig. S7. The predicted persuasiveness was elicited on a comprehensive numerical scale ranging from −10 to +10 (58).

#### Step 4: testing the predicted persuasiveness

As a final step, we conducted a preregistered randomized survey experiment to empirically assess whether the predicted persuasiveness gathered in step 3 aligns with the actual persuasiveness when readers hold baseline beliefs against climate change. The experiment was conducted on Prolific with 1,000 US participants, all prescreened to be climate skeptics. The distribution of respondents climate stance can be seen in Fig. S1.

Through random assignment, one-third of participants was allocated to a control group, while two thirds to a treatment group. Participants in the treatment group read one of the 60 articles about climate change. Participants in the control group read one of two possible control articles, which were selected to be benign and unrelated to climate change topic. Specifically, the control group articles covered topics such as space exploration and the importance of investment in education. For further details, refer to Supplementary Material section, Research design and crowdsourced tasks.

After reading the assigned article, participants were prompted to answer a set of 12 questions designed to assess their attitudes toward climate change. These questions spanned three distinct dimensions: beliefs in climate change, endorsement of climate policies, and intentions to undertake personal actions contributing to climate change mitigation (59). Subsequently, participants were invited to allocate a flexible portion of their bonus to a nongovernmental organization (NGO), with the option to choose an NGO either in opposition to or in support of climate policies.

Finally, we elicited the emotional response to news articles on a set of ten positive and negative emotions following the International Positive and Negative Affect Schedule Short Form (I-PANAS-SF) scale (60).

## Results

In this section, we examine the primary findings from steps 3 and 4 described in Materials and methods and illustrated in Fig 1. Our goal is to compare and contrast the predicted and actual persuasiveness of everyday news articles about climate change. When presenting the results, we classify both the forecasters and the target readers into three groups according to their stance on climate change: skeptics, moderates, and advocates (see Supplementary Material section, The climate stance index for specific group definitions).

### Predicted effects

Herein, we introduce the results of the prediction survey (step 3 in Fig. 1), first at the article level and then at an aggregated level.

#### Predictions at the article level

**Fig. 2.**
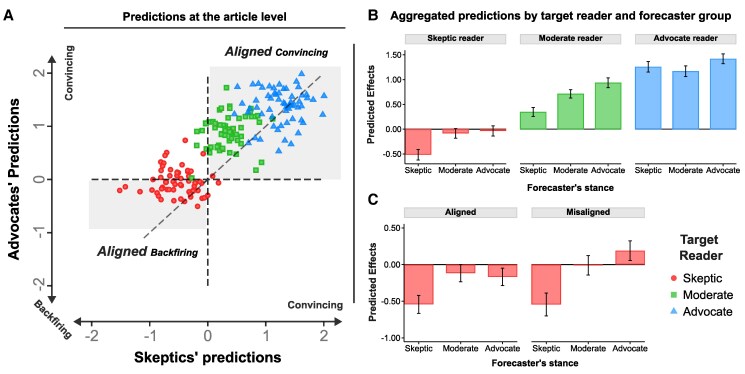
Predicted effects on climate change attitudes, by baseline climate stance of forecasters and target readers. Panel A) examines the alignment of the predictions of skeptics and advocates at the article level. Each article is depicted three times in different shapes, corresponding to different target reader groups for whom the predictions are being made (skeptics as circle, moderates as square, and advocates as triangle). The *x*-axis represents the average predictions made by skeptic forecasters, while the *y*-axis represents the average predictions made by advocates. The highlighted quadrants denote articles for which both skeptic and advocate forecasters agree on the sign of the predicted persuasion effect. The dashed diagonal line represents the identity line y=x, with articles closer to it indicating stronger agreement in the magnitude and direction of the predicted effect. Panel B) displays the average predictions per target reader group, by forecaster’s stance; the effects are estimated by regressing the predicted change for each reader category on dummy variables for each type of forecaster. Panel C) disaggregates the average predictions for skeptic readers, distinguishing between sets of articles where the predictions of skeptic and advocate forecasters either align or diverge. In all panels, the error bars represent 95% CIs of the means; all predicted effects are obtained by standardizing the response by the SD within the target group category, and SEs are clustered at the respondent level. The figure shows that persuasion predictions differ based on the climate stance of the forecaster and the target reader. For skeptic readers, skeptic forecasters predict a backfire effect, while moderates and advocates predict no effect. This diversity in perspectives prompted us to conduct a follow-up experiment to empirically assess the actual persuasiveness and compare it with these forecasts.

In Fig. 2A, we show the distribution of predictions for the 60 most persuasive articles. The *x*-axis represents the average predictions made by skeptics, while the *y*-axis represents those made by advocates (comparisons across other forecaster groups are presented in Fig. S8). All effect sizes are normalized by the SD in the corresponding group. Each article is depicted three times on the plot—once for each target reader group—distinguished by different colors and shapes. The gray quadrants indicate articles for which both skeptic and advocate forecasters agree, on average, on the direction of the predicted effect. The diagonal gray line represents the y=x axis: points closer to this line signal higher agreement, while those farther away indicate higher levels of disagreement; the white quadrants indicate articles for which predictions are misaligned.

The majority of the articles in our sample fall into either of the two aligned quadrants, although a sizable share does not. In particular, for the target group of skeptics (represented by the green circles), slightly more than one-third of articles fall into a misaligned quadrant. Notably, skeptic forecasters consistently predict a backfire effect for like-minded individuals, while advocates show considerable heterogeneity in their predictions.

#### Aggregated predictions by target and forecaster group

In Fig. 2B, we depict the average predicted effects, stratified by the climate stance of both forecasters and the target reader group. Our analysis reveals a prevailing optimism regarding the persuasiveness of climate change articles for readers already moderately or strongly concerned with climate issues. However, when it comes to skeptic readers, this optimism dissipates and forecasters do not anticipate the articles being persuasive.

More precisely, for the advocate reader group the effect sizes stand at +1.26, +1.17, and +1.42 SDs for predictions made by skeptic, moderate, and advocate forecasters, respectively, (P<0.001). Additionally, the predicted magnitude by advocate forecasters significantly exceeds that of moderate (P<0.001, $\chi^{2}$ test) and skeptic (P=0.03) forecasters. Note that all SEs are computed by clustering the responses at the respondent level. Additionally, *P*-values are calculated using a two-sided t test with the corresponding SEs, unless specified otherwise.

For the moderate reader group, there is qualitative agreement across forecasters regarding an expected increase in support for climate actions, however, we observe disparities regarding the magnitude of this anticipated increase. Advocates express the highest degree of optimism (effect of +0.94 SDs, P<0.001), while moderates hold a more tempered expectation (+0.71, P<0.001) and skeptics anticipate the smallest effect size (+0.35, P<0.001). All differences in expected magnitudes are statistically significant (P<0.001).

For the skeptic reader group, moderate and advocate forecasters, on average, anticipate little or no persuasion. Effect sizes range from −0.09 SDs (P=0.08) for moderate forecasters to −0.04 SDs (P=0.48) for advocate forecasters. Conversely, skeptics foresee a significant backfire effect, where exposure to these articles intensifies opposition to climate policies among skeptic readers. Namely, skeptics anticipate an effect size of −0.52 SDs (P<0.001). Moreover, the magnitude of the average predicted effect by skeptics significantly differs from the average predictions of both moderates and advocates (P<0.001 in $\chi^{2}$ tests comparing skeptics versus moderates and skeptics versus advocates). This underscores the distinct perspective held by skeptics, indicating a divergence in their expectations compared with both moderates and advocates.

#### Aggregated predictions for skeptic readers

In total, we observe a set of 35 articles for which the predictions are aligned, whereby both skeptic and advocate forecasters anticipate a *backfire effect* from skeptic readers.

Among the remaining articles, we identify 21 for which the predictions of skeptics and advocates are misaligned. While skeptics predict a backfire effect of −0.54 (P<0.001), advocates predict that these articles will, on average, be persuasive, with an effect size of +0.19 (P=0.05). The difference in the average predictions is −0.73 SDs (P<0.001 in a $\chi^{2}$ test). For an illustration of these results, refer to Fig. 2C.^c^

#### Summary

In summary, forecasters anticipate significant and relatively robust persuasion effects from climate change articles on the attitudes of moderate and advocate readers. However, a prevailing sense of pessimism emerges regarding the expected impact on skeptic readers. Advocate forecasters foresee no discernible effects, while skeptic forecasters anticipate a notably large backfire effect. Given the pessimistic outlook on persuading climate skeptics, it is crucial to empirically verify whether these predictions hold true and to compare the expected effects of reading newspaper articles with the actual observed outcomes.

### Actual effects

Next, we present the actual observed changes in the attitudes of skeptics following their exposure to newspaper articles on climate change. To this aim, we analyze the data collected during the randomized survey experiment (step 4 in Fig. 1). In this phase, a total of 1,000 skeptics were randomly assigned to read one of the 60 preselected articles on climate change or one of the two control articles. After implementing our preregistered exclusion criteria and excluding additional responses with missing data on key outcomes, our final sample comprises 935 observations. Supplementary Material section, The econometric analysis section provides details on the estimated regression models.

#### Average treatment effects

**Fig. 3.**
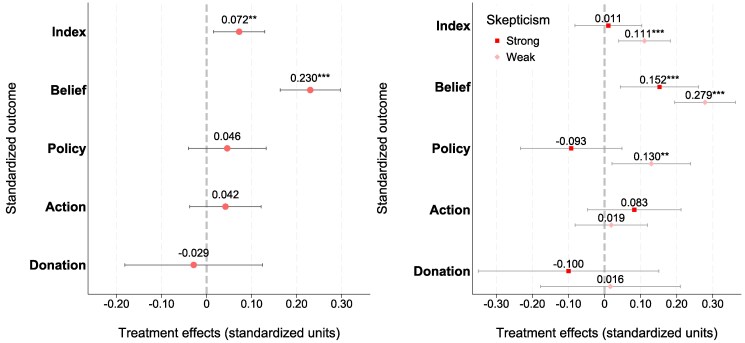
Average treatment effects on skeptics’ attitude towards climate change. Panel A) presents average treatment effects in the entire sample. Panel B) presents marginal treatment effects, distinguishing among strong and weak skeptic readers. The outcome variables are standardized indices capturing four climate support components: beliefs about the severity of climate change, support for climate policy, intention to adopt private mitigation actions, and actual donations to NGOs that are either supportive or against climate action. Additionally, the Index represents an average that equally weighs the four climate support components. The estimated models control for respondents’ baseline climate change beliefs and socio-demographics; error bars represent 95% CIs around the estimated means. Panel (A) demonstrates that the treatment significantly enhances readers’ overall attitudes toward climate change, as captured by the Index. This effect is primarily driven by an increase in concerns about climate change, reflected in the belief component. Panel (B) highlights that these effects are more pronounced among weak skeptics compared with strong skeptics. Statistical significance: *** P<0.01, ** P<0.05, and * P<0.1. Panels (A): average treatment effects and (B): marginal treatment effects.

Fig. 3A illustrates the average treatment effect of reading a climate change article relative to reading a control article. We study four attitude outcomes: beliefs, policy support, actions, and NGO donation. Moreover, to examine aggregate effects, we construct an index that equally weighs the four attitude outcomes. We find a positive and significant persuasion effect, measured as an increase of 0.072 SDs in the general attitude index (P=0.013). When analyzing changes in individual attitudes, it appears clear that the overall persuasion effect is driven by a shift in beliefs regarding the urgency of climate change (an increase by 0.230 SDs, P<0.001). Importantly, this effect size, when compared with analogous studies on persuasion experiments, demonstrates not only statistical significance but also considerable magnitude. For instance, in the meta analysis by Rode et al. (43), an effect size of g=0.08 is found by analyzing 396 effect sizes derived from 76 distinct experiments. These findings remain robust when estimating article-level random effects models (see Panel A in Figs. S16–S17).

Despite the significant persuasion of beliefs, we find that respondents’ policy support, actions, and donations remain unaffected by the treatment (see Figs. S9–S12 for estimated average treatment effects on the individual components of these outcomes). These findings resonate with existing research on climate change persuasion, highlighting the heightened resistance to influence in the domain of policy attitudes and individual behavior (43, 45).

#### Heterogeneity

To understand the effects on our outcome variables at a deeper level, we study treatment effect heterogeneity, distinguishing respondents according to the strength of their baseline skepticism to climate change (not preregistered). Namely, we separate skeptics into two groups of strong and weak skeptics based on their responses to climate questions (details of this classification can be found in Supplementary Material section, Research design and crowdsourced tasks). Figure 3B displays the estimated marginal treatment effects from regression models where the treatment is interacted with an indicator for strong skepticism. The analysis reveals heterogeneity in the impact of reading climate change articles: while among strong skeptics the overall attitude index remains unchanged, a positive and significant persuasion effect is observed among weak skeptics (an effect size of +0.11 SDs, P=0.003).

Furthermore, the treatment significantly increases the belief and policy support scores of weak skeptics (effect sizes of 0.28 SDs, P<0.001, and 1.3 SDs, P=0.02, respectively). In contrast, treatment effects on beliefs and policy support are significantly lower among strong skeptics, with a marginal treatment effect on the belief score of about +1.15 SDs (P=0.006) and no significant effects on policy support. Additionally, we test the robustness of this specification by estimating an interaction model where the treatment indicator is interacted with a continuous measure of respondents’ pretreatment climate stance, and the results are presented in Table S5.

#### Summary

In summary, we find a significant positive persuasion effect for climate skeptics, driven by the impact on skeptics belief about climate change. Furthermore, the heterogeneity analysis highlights two key implications. First, when baseline skepticism is relatively weaker, a simple exposure to news articles about climate change can be effectively persuasive and induce both beliefs shifts and enhance policy support. Second, among strong skeptics, attitudes are significantly less malleable. Nonetheless, it is noteworthy that even among strong skeptics, there exists a potential, albeit limited, for persuasion in shaping their beliefs.

### Predicted versus actual persuasion

So far, we have documented the existence of a big rift between the predicted attitude change and the actual impact on climate skeptics. On the one hand, we found a prevailing pessimism in predictions, the degree of which varies in accordance to forecasters’ climate stance; precisely, skeptics anticipate a backfire effect among their peers, while advocates foresee no substantial changes among skeptics’ attitudes. On the other hand, upon examining actual effects, the treatment proves successful in significantly persuading skeptics’ overall attitudes towards climate change. Herein, we offer additional insights into the discrepancies between individuals’ expectations and the real effects of exposure to climate-related news articles.

#### Article-level effects

**Fig. 4.**
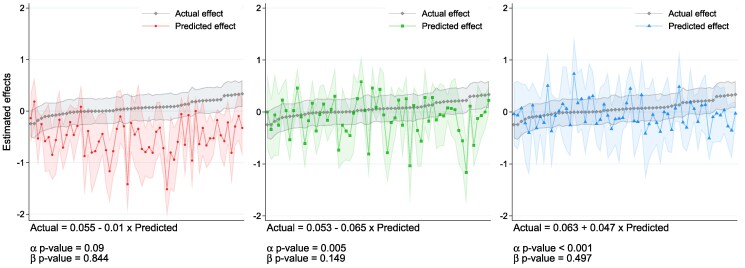
Predicted versus actual effects. In each panel, the actual average effects and their 95% CIs are depicted in gray. The average article-level predictions are depicted in Panel A) when forecasters are skeptics, in Panel B) when forecasters are moderates, and in Panel C) when forecasters are advocates. The actual treatment effects for each article are estimated using OLS to fit the following model: $Y_{i}=\sum_{k=1}^{K}\beta_{k}I_{k}+X_{i}^{\prime }\Gamma +\epsilon_{i}$, where $Y_{i}$ is the index of climate support of the skeptic reader *i*, K=60 is the number of treatment articles, and $I_{k}$ are dummy indicator for each treatment article. $X_{i}$ is a vector of individual characteristics, and $\epsilon_{i}$ is the error term. To estimate the average predictions, we estimate the following model: $P_{j}=\sum_{k=1}^{K}\beta_{k}I_{k}+\epsilon_{j}$, where $P_{j}$ is the prediction on the impact of article *k* by forecaster *j*. We distinguish between skeptic, moderate, and advocate forecasters. Standard errors are clustered at the forecaster level. The figure shows that forecasters consistently underestimate actual effects, as indicated by positive and significant intercepts, while the variation in actual effects is poorly captured by predictions, with beta coefficients near zero and statistically insignificant across all climate stances. Panels (A): predictions by skeptics, (B): predictions by moderates and (C): predictions by advocates.

Figure 4 illustrates the article-level average effects (aggregated index) along with their 95% CIs depicted in gray. The *x*-axis displays the coefficients for each of the 60 articles arranged in ascending order based on the magnitude of actual effects. In color, the figure portrays the estimated average *predicted changes* for each article, revealing notable volatility relative to the actual effects. Additionally, the figure presents the estimated coefficients of simple linear regression models, where actual effects are regressed on a constant and the predicted effects. The *P*-values for both coefficients are shown below the equation.

Our findings indicate that forecasters, regardless of their stance, tend to underestimate the actual effects, as evidenced by the positive and statistically significant intercepts in the ordinary least squares models. Moreover, the variation in actual effects cannot be adequately captured by the predictions, as shown by beta coefficients that are close to zero and not statistically significant across all climate stances. A similar analysis indicates that both weak and strong skeptics significantly underestimate the actual effects, although the degree of underestimation is lower among weak skeptics compared with strong skeptics (see Fig. S18).

#### Aligned and misaligned predictions

Next, we examine heterogeneity for the two subsets of articles where skeptics’ and advocates’ predictions were either aligned or misaligned. For a set of 35 articles (the *aligned* set), both skeptics and advocates on average predict a backfire effect. In contrast, for a set of 21 articles, advocates expect persuasion, while skeptics predict backfire.

**Fig. 5.**
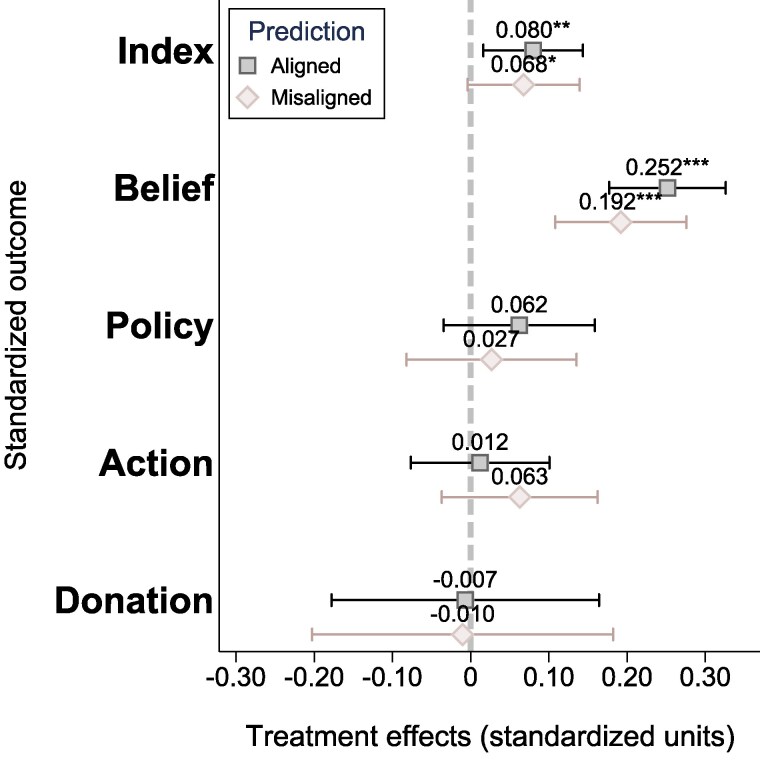
Treatment effects for aligned and misaligned articles. To estimate the model, treatment articles are classified based on the alignment in predictions by skeptic and advocate forecasters (see Results section). In our sample, we observe 35 “aligned” articles, 21 “misaligned” articles, 4 “neither aligned nor misaligned” articles, and 2 control articles. To improve visibility, we omit reporting the coefficients for the “neither aligned nor misaligned” articles, although they have been included in the analysis. The estimated models control for respondents’ baseline climate change beliefs and socio-demographics; the error bars represent 95% CIs of the means. The figure shows that both aligned and misaligned article sets are persuasive for the overall attitude index, driven by changes in beliefs. Statistical significance: *** P<0.01, ** P<0.05, and * P<0.1.

Figure 5 presents the estimated treatment effects of the two sets of articles on the attitude outcomes of climate skeptics. Both sets of articles appear persuasive with respect to the overall attitude index, an effect driven primarily by changes in beliefs. Moreover, the effect sizes have similar magnitudes, as indicated by the largely overlapping CIs. For more details on the analysis and formal tests of coefficient equality, see Table S4. The *aligned* coefficient underscores the limited ability of both skeptics and advocates in predicting the persuasive impact of news articles, as this is the set for which both groups predicted a backfire effect. Nevertheless, the notable positive and significant effect of *misaligned* articles suggests once more a relatively more accurate prediction ability among advocates, as their prediction is in the same direction of the actual effect.

#### Summary

Overall, our findings uncover a significant divergence between predicted outcomes and observed impacts. Particularly striking is the stark contrast between the average prediction of skeptics and the actual effect observed, which contradicts the hypothesis that skeptics have an advantage in accurately predicting opinion shifts of their like-minded peers (*H1*). Furthermore, the significant increase in the overall attitude index appears to elude advocates’ predictions of no change as well. Yet, their predictions came closer to the actual effects, at least with respect to shifts in policy support, actions, and donations.

### Emotional response

Finally, we estimate the treatment effects on participants’ self-reported emotional responses provided at the end of the survey. As outcome variables, we construct two standardized indices, one for negative and one for positive emotions; additionally, we consider the difference between negative and positive emotions.

**Fig. 6.**
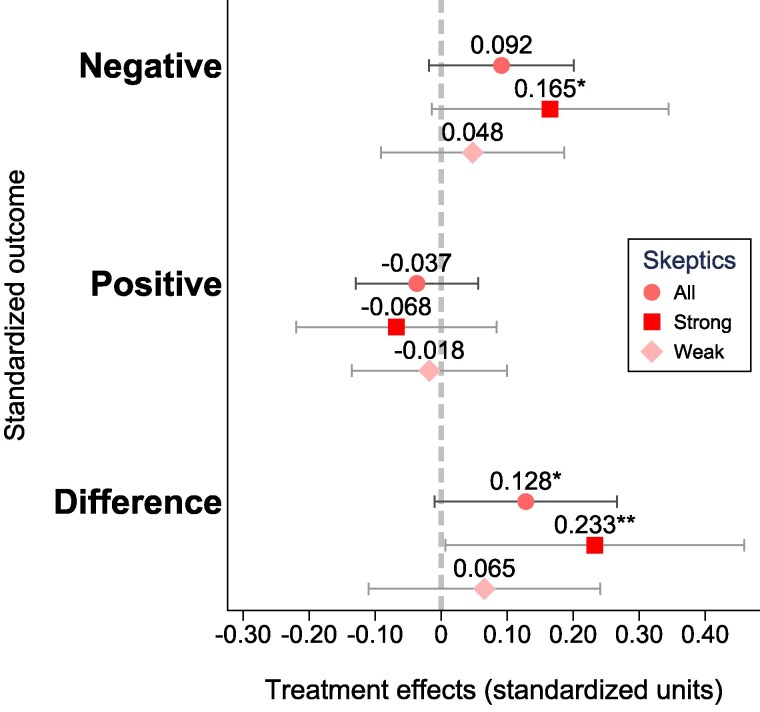
Average treatment effects on skeptic readers’ emotions. The outcome variables are indices of negative and positive emotions, and the difference between the negative and positive indices. The emotions were measured using the International Positive and Negative Affect Schedule Short Form (I-PANAS-SF) scale (60). The estimated models control for respondents’ baseline climate change beliefs and socio-demographics; the error bars represent 95% CIs around the estimated means. While the “all” coefficients represent average treatment effects in the entire sample, the “strong” and “weak” coefficients represent marginal treatment effects, distinguishing among strong and weak skeptic readers based on their baseline climate beliefs and support for climate action. The figure shows that reading articles emphasizing the severity of climate change leads to a heightened difference between negative and positive emotional reactions, with this effect being particularly pronounced among strong skeptics. Statistical significance: *** P<0.01, ** P<0.05, and * P<0.1.

Figure 6 presents the estimated effects for the entire sample of participants, as well as marginal treatment effects for the subsets of strong and weak skeptics. Overall, the aggregate sample shows a weak emotional response, with neither positive nor negative emotions significantly impacted. For weak skeptics, we observe no significant changes in emotions, with effect sizes close to zero. In contrast, among strong skeptics, we find a slight increase in negative emotions (+0.17 SDs, P=0.071) and a corresponding increase in the disparity between negative and positive emotional scores (+0.23 SDs, P=0.044). Figs. S14–S15 present the estimated treatment effects for each of the 10 emotions.

#### Summary

Contrary to expectations, exposing climate skeptics to information about climate change—thereby challenging their baseline beliefs—did not elicit a strong emotional response. We find no support for *H2* in the aggregate sample, as neither positive nor negative emotions were significantly impacted. This lack of a strong emotional reaction—which matches the low sentiment scores of the articles selected for the experiment (see Supplementary Material section Sentiment of news articles)—may have allowed for belief updating, which strong negative emotions might have prevented.

However, we observe some emotional response among strong skeptics, providing weak support for *H2* within this group. Specifically, strong skeptics, whose climate concerns reacted less to the treatment compared with weak skeptics, displayed mild negative emotional reactions. This pattern suggests that negative emotions in response to climate change articles grow stronger with higher levels of skepticism and may hinder belief updating, but only when skepticism is deeply entrenched.

## Discussion

Climate communication efforts targeting skeptics critically hinge on the expectation of skeptics’ responsiveness to climate messages; in this context, misperceptions can fatally erode cooperation and prevent collective climate action altogether (21).

In this study, we elicited and tested predictions about the persuasiveness of climate news articles on climate skeptics’ attitudes through a comprehensive series of online surveys with respondents from the United States. To our knowledge, this is the first study to investigate persuasion expectations stratified by the climate stance of forecasters in the context of climate change. Our findings reveal a striking divergence between anticipated and actual effects of climate communication targeting skeptics. While predictions are broadly pessimistic—with advocates and moderates anticipating no effect and skeptics expecting a backfire—our results provide robust evidence that exposure to climate news articles significantly shifts skeptics’ attitudes in a positive direction.

However, while skeptics are persuaded to reevaluate the severity of climate change, this attitudinal shift does not translate into increased support for climate policies or proenvironmental behaviors. These results highlight an attitude–behavior gap, where changes in beliefs do not consistently lead to action, as documented by prior research (43, 45).

The observed attitude–behavior gap in our study raises questions about its underlying causes, offering avenues for future research. On the one hand, the climate articles predominantly focused on the impacts and severity of climate change, with little emphasis on specific policies or individual actions to address the issue. As a result, while the articles may have heightened concern about climate change, they likely did not empower readers to act, leaving their policy support, proenvironmental behavior, and donations unchanged. This argument is consistent with findings by (44), who highlighted the “awareness-action inconsistency,” noting that increased climate concern does not automatically lead to behavioral change without addressing underlying psychological and contextual factors. Future experimental designs could test whether incorporating actionable practices alongside climate change information reduces the attitude–behavior gap.

On the other hand, the persistence of the gap may suggest that misinformation about climate change is not the primary barrier to action among skeptics. Instead, it may reflect a deliberate choice to align with preferences for inaction. This perspective is consistent with (49), who, in the context of immigration, posits that misperceptions about the size of minority groups may be a consequence, rather than a cause, of attitudes toward those groups. Similarly, in the context of climate change, misinformation may serve as a justification for preexisting preferences, rather than being a root cause of inaction. Future research could explore this possibility to better understand the barriers to addressing climate change among skeptics.

Our findings challenge the hypothesis that members of an ingroup are better equipped to predict the behavior of their peers, as supported by theories such as social identity theory and ingroup empathy, which argue that shared group membership enhances perspective taking. In the context of climate change, we find no evidence that climate skeptics are better at predicting the persuasion of fellow skeptics. Instead, shared skepticism appears to hinder accurate forecasting, potentially due to distorted perceptions of ingroup norms—a mechanism suggested by You and Lee (53) in the context of affective polarization within political partisanship. This proposed mechanism aligns with the findings of Andre et al. (61), which demonstrate the effectiveness of correcting misperceptions about the prevalence of climate-friendly behavior in the US population in increasing mitigation efforts, particularly among skeptics. Nevertheless, the exact underlying mechanism, as well as potential alternative explanations, require further investigation in future research.

Our results carry important societal and policy implications. In social contexts, the motivation to share news often stems from a desire to influence others’ perspectives (62). Consequently, individuals’ expectations regarding the potential for opinion change play a crucial role in guiding their decision-making processes related to information sharing. These expectations are formed based on previous experiences, perceived credibility of the information, and the anticipated receptiveness of the target audience. Accordingly, overly pessimistic expectations could intensify existing polarization by discouraging individuals from sharing potentially useful information. Our findings reveal that, on average, skeptics’ opinions are more malleable than previously anticipated. This insight can foster more constructive dialogue on this topic, encouraging those people who would normally abstain from cross-partisan interactions for fears of conflict (25, 26) to engage more openly and effectively.

Furthermore, our results challenge prevailing pessimism by demonstrating that climate skeptics can indeed be persuaded about the severity of climate change. The settings of our experiment give us hope that our results are relevant also in real-world scenarios for at least two reasons. First, persuasion was observed after a single exposure to an everyday news article about climate change. Second, because the articles used in our experiment are short, do not require specialized background knowledge, and are not ideologically loaded, they could be found in the news feeds of social media users regardless of their political stance. Furthermore, following recent insights from the political science literature, focusing on dyadic communication between users that share common nonpolarizing features could yield even larger impacts (63, 64).

We note some limitations of our study. Specifically, we did not reveal the source of the article to the readers, unlike real-world scenarios where people are more inclined to trust news from familiar sources. This design choice was intentional to exclude the impact of source credibility and to measure the content’s impact in a controlled setting. As a result, this could potentially lead to larger effect sizes than what might be observed in real life, as readers may have approached the content more open-mindedly without an ideological slant guiding their biases. Furthermore, our experiment involved exposing skeptics to news articles on climate change, which may not always reflect real-world information consumption patterns where individuals often seek out sources that align with their existing beliefs (12, 13). This highlights the need for nuanced approaches in climate science communication to address information silos and echo chambers. Finally, there is a possibility that experimenter demand effects contributed to the observed changes in attitudes among climate skeptics; however, we provide a detailed discussion in the Supplementary Materials, offering both design-based and results-based arguments that minimize these concerns (see Supplementary Material section Experimenter demand effect, and Figs. S13, S19, and S20).

The path to effective bipartisan cooperation on climate action contains a fork with two branches that must be navigated in parallel. The first branch, which we examined in this paper, is about aligning perceptions of how climate skeptics react to climate information with reality. As with other polarized issues, cross-partisan views on climate change are closer than they initially appear and may even be reconcilable (65). However, this alone is not sufficient. The second branch requires understanding how individuals self-select climate information and designing strategies to penetrate echo chambers with climate communication. A top-down strategy could involve altering the news feed algorithms (66), though this approach is not without controversy; a bottom-up strategy might promote media literacy and critical thinking skills to encourage individuals to engage with diverse perspectives and evidence-based information (67), even when it challenges their existing beliefs. By acknowledging and addressing the complexity of climate communication, we can tailor efforts to effectively engage skeptics and foster an informed dialogue on climate change.

## Supplementary Material

pgaf084_Supplementary_Data

## Data Availability

All newspaper articles used in this study were accessed through a data-sharing agreement with Factiva. Other researchers can obtain a similar agreement to access the articles. The newspaper articles used for the survey experiment are available on the OSF project page: https://rb.gy/kj8ty. Additionally, the survey data collected for this study, along with the analysis code—including scripts for generating figures and tables presented in this paper—are openly accessible on the same OSF project page.

## Notes

a See: https://aspredicted.org/67B_RVL; more details are available at OSF: https://rb.gy/kj8ty.

b See: https://professional.dowjones.com/factiva/

c The remaining set of four articles that do not correspond to either the aligned or misaligned categories are labeled as “Other” and included in the analysis as a standalone category; for clarity of exposition, we omit reporting these coefficients here.
