# Supplementary material for: Divergence between predicted and actual perception of climate information
Source: PNAS Nexus. 2025 Mar 18;4(3):pgaf084. doi: 10.1093/pnasnexus/pgaf084 (PMC11935527; doi:10.1093/pnasnexus/pgaf084)
Supplement: pgaf084_Supplementary_Data [file pgaf084_supplementary_data.pdf]

Supplementary Materials

Contents

1 Research Design and Crowdsourced Tasks **1**
- 1.1 **The Climate Stance Index** 1
  - 1.1.1 Strong and weak skeptics 1
- 1.2 **Survey Step 2: Broad evaluation for pertinence and persuasiveness** 1
  - 1.2.1 *Structure* 1
  - 1.2.2 *Quality controls and training* 1
  - 1.2.3 *Source and publication year of articles* 2
  - 1.2.4 *The labeling task* 2
  - 1.2.5 *Respondent characteristics* 4
  - 1.2.6 *Average time and pay* 4
- 1.3 **Survey Step 3: Predicting persuasiveness** 4
  - 1.3.1 *Article selection* 4
  - 1.3.2 *Thematic analysis* 5
  - 1.3.3 *Structure* 5
  - 1.3.4 *Respondent characteristics* 5
  - 1.3.5 *Average time and pay* 6
- 1.4 **Randomized Experiment Step 4: Testing predicted persuasiveness** 6
  - 1.4.1 *Structure* 6
  - 1.4.2 *Outcome measurement* 7
  - 1.4.3 *Respondent characteristics* 7
  - 1.4.4 *Average time and pay* 8
  - 1.4.5 *Average predictions by forecaster's stance* 8

2 The Econometric Analysis **8**
- 2.1 **Aggregate treatment effects** 8
- 2.2 **Alignment and misalignment in predictions** 9
- 2.3 **Heterogeneous treatment effects by pre-treatment climate skepticism** 9
- 2.4 **Emotions** 11
- 2.5 **Predicted versus actual effects** 11
- 2.6 **Robustness checks** 11

3 Experimenter Demand Effect **11**
- 3.1 **Insights from open-ended feedback** 13

4 Sentiment of News Articles **14**

Research Design and Crowdsourced Tasks

Here, we provide additional details on the experimental procedures illustrated in Fig. 1. Further information, including the full set of survey questions and all articles used in the experiment, is available on the project's OSF page at `https://rb.gy/kj8ty`. Additionally, all data and codes used for analysis and the generation of figures and tables are openly accessible at the same OSF project page.

The Climate Stance Index

In all crowdsourced tasks, we quantified the respondents' stance on climate change and categorized them into three distinct groups: Skeptics, Moderates, and Advocates. The categorization procedure relied on a modified version of the method developed by [6], where we aggregated responses to the following five questions probing participants' views on climate change and environmental issues:[1]

*Q*1. I consider myself an environmentalist.
*Q*2. I believe that man-made climate change is occurring.
*Q*3. The United States was right to rejoin the Paris Agreement in 2021 to reduce greenhouse gas emissions.
*Q*4. In general, how much do you trust the science on global warming?
*Q*5. How worried are you about climate change?

Each question was rated on a five-point scale, resulting in a Climate Stance index ranging from 5 to 25. Based on this score, individuals were classified into three groups: those scoring below 15 were identified as *Skeptics*, those with scores exceeding 20 were classified as *Advocates*, and respondents with scores between these two extremes were designated as *Moderates*. The rationale for these thresholds is derived from the structure of the index. Respondents classified as skeptics have an average response score of less than 3, while advocates have an average response score of 4 or higher. Consequently, when posed with the questions above, skeptics typically fall within the "strongly disagree" to "disagre" range, while advocates fall within the "agree" to "strongly agree" range. The distribution of respondents' Climate Stance Index is presented in Figure S1.

Strong and weak skeptics
We further classify skeptics into two categories: strong skeptics, defined as individuals with a Climate Stance Index below 10, and weak skeptics, identified as those with a Climate Stance Index between 10 and 15. The right panel in Figure S1 shows the distribution of Climate Stance Index scores by strong and weak skepticism.

Survey Step 2: Broad evaluation for pertinence and persuasiveness

Structure
In the initial stages of this survey, respondents were queried on a range of demographic factors, such as age, educational attainment, and gender, enabling a detailed understanding of their backgrounds. This was followed by inquiries concerning their political orientation. Subsequently, we assessed their viewpoints on various aspects of climate change through a series of targeted questions, evaluating their level of concern, acceptance of anthropogenic climate change, and confidence in climate science.

Quality controls and training
To validate respondents' ability to comprehend and critically evaluate news articles, we incorporated different comprehension checks. Both as a screening and as a training, three exemplary articles were presented in random order: one supportive of climate change actions, one denying the existence of climate change, and one unrelated to the subject matter. Respondents were required to successfully identify which article tackled the issue of climate change before proceeding to assess a randomly selected set of 12 articles. Within this set, two articles (the 2nd and the 11th) were fixed for all participants and their labeling served as attention checks, facilitating the identification of

[1] In Q1-Q3, respondents were asked to express their agreement or disagreement with the presented statement. In Q4-Q5, answers were elicited on a 5-point Likert scale.

Draft

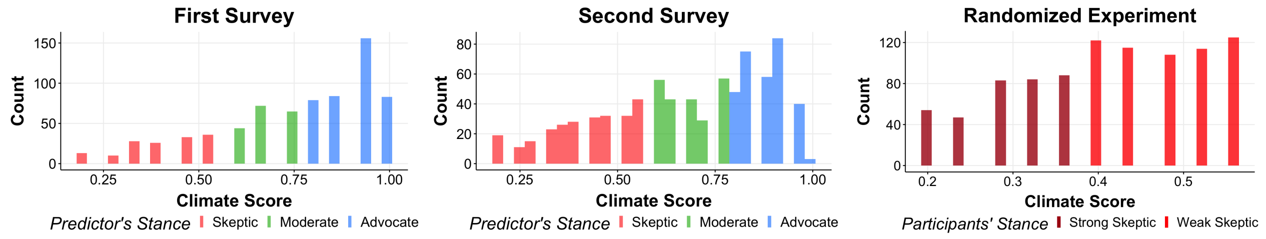

Figure S1. Distribution of climate scores of the participants in the first survey (left), second survey (middle) and the randomized experiment (right). The cut-off used in each survey is illustrated by the colors.

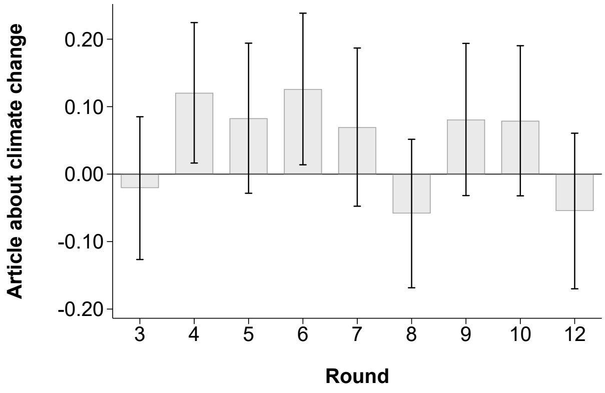

Figure S2. Share of participants saying article is covering the topic of climate change, in each round relative to round 1. Estimates are obtained by regressing an indicator of whether the article was labeled as being on the topic of climate change on round fixed effects, incorporating respondent fixed effects and clustering errors at the respondent level. The base category for the round fixed effects is round 1. Rounds 2 and 11 are removed from the figure, as they were attention checks with fixed articles.

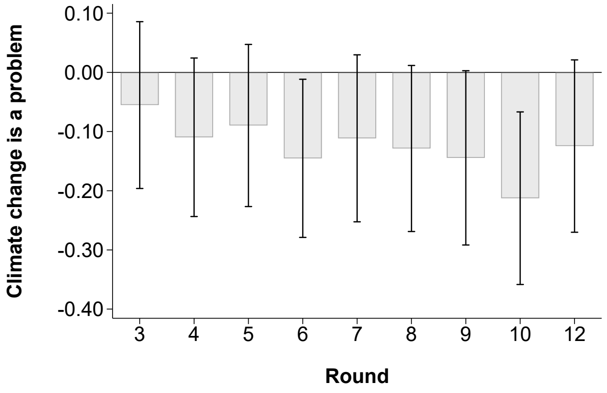

Figure S3. Average score assigned to article in the question whether climate change is a problem (on a 5-point Likert scale), in each round relative to round 1. Estimates are obtained by regressing a categorical variable (values 1-5) capturing the severity of the climate change problem on round fixed effects, incorporating respondent fixed effects and clustering errors at the respondent level. The base category for the round fixed effects is round 1. Rounds 2 and 11 are removed from the figure, as they were attention checks with fixed articles.

respondents whose accuracy may diminish over the course of the survey. The order of the remaining 10 articles was randomized.

Figures S2 and S3 demonstrate strong consistency in participants' labeling of articles across rounds, both in identifying whether the article addresses the topic of climate change and in assessing the stance of the article regarding the severity of the problem depicted. The results indicate an absence of spillover effects from labeling one article on the labeling of subsequent articles. Article number 2 and 11 are removed from the figure, as they were fixed and not randomized.

Source and publication year of articles

The sample consisted of articles from a very diverse set of publishers. In total, there were 654 unique sources, most of which had very few articles. Table S2 shows the name of top publishers which had at least 10 articles within our sample. Note that the name of source was not shown to the readers and only the title and publishing date were disclosed. Figure S4 shows the distribution of publication years for the 1,350 articles utilized in the initial survey task. As it can be seen, most of the articles are published in more recent years.

The labeling task

For each of the 12 articles, respondents were first tasked with determining whether the article predominantly addressed

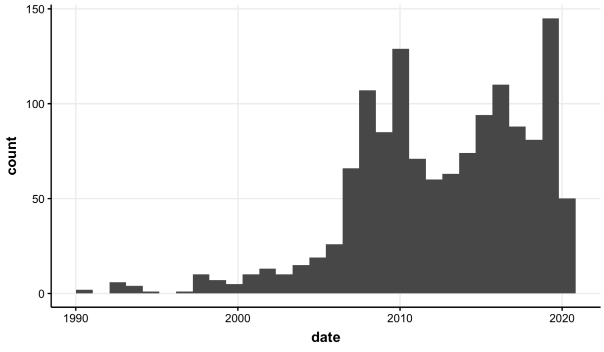

Figure S4. Distribution of publication years for the 1,350 articles utilized in the initial survey task.

the topic of climate change. If their response was negative, they were directed to the subsequent article. If their response was affirmative, additional answers were gathered concerning the article's stance on the gravity of climate change, its argumentative style (e.g., reliance on empirical data and/or statistics or anecdotal narratives), and whether the information was perceived as deceptive or misleading.

Table S1. Characteristics of respondents involved in all three surveys. The *Liberal vs Conservative* variable is rated on a scale from 1 (Strongly Liberal) to 7 (Strongly Conservative). The *Following Politics* variable indicates how many days a week they follow politics.

Characteristic	Value	Broad Search (N = 777)	Narrow Filter (N = 796)	Experiment (N = 935)
**Age**				
	Median (IQR)	37 (28, 52)	42 (32, 54)	43 (32, 54)
**Education**				
	College	398 (51%)	365 (46%)	477 (51%)
	Elementary	3 (0.4%)	11 (1.4%)	10 (1.1%)
	Grad School	131 (17%)	140 (18%)	117 (12%)
	High School	245 (32%)	279 (35%)	330 (35%)
**Race**				
	African American	36 (4.6%)	73 (9.2%)	57 (6.1%)
	African American, American Indian, White	4 (0.5%)	2 (0.3%)	-
	African American, Latino	1 (0.1%)	-	-
	African American, Native Hawaiian, White	1 (0.1%)	-	-
	African American, White	6 (0.8%)	10 (1.3%)	-
	American Indian	1 (0.1%)	2 (0.3%)	23 (2.5%)
	American Indian, Asian, White	2 (0.3%)	-	-
	American Indian, Latino, White	1 (0.1%)	-	-
	American Indian, White	9 (1.2%)	5 (0.6%)	-
	Asian	37 (4.8%)	36 (4.5%)	38 (4.0%)
	Asian, Latino	1 (0.1%)	3 (0.4%)	-
	Asian, Latino, White	1 (0.1%)	1 (0.1%)	-
	Asian, Native Hawaiian	1 (0.1%)	-	-
	Asian, Pacific Islander, White	2 (0.3%)	-	-
	Asian, White	15 (1.9%)	4 (0.5%)	-
	Latino	26 (3.3%)	19 (2.4%)	63 (6.7%)
	Latino, White	24 (3.1%)	22 (2.8%)	-
	Native Hawaiian, Pacific Islander	1 (0.1%)	1 (0.1%)	1 (0.1%)
	White	608 (78%)	611 (77%)	753 (81%)
**Gender**				
	Female	404 (52%)	400 (50%)	476 (51%)
	Male	357 (46%)	385 (48%)	452 (48%)
	Non-binary	16 (2.1%)	11 (1.4%)	7 (0.7%)
**Liberal vs Conservative**				
	1	179 (23%)	112 (14%)	8 (0.9%)
	2	155 (20%)	99 (12%)	13 (1.4%)
	3	50 (6.4%)	96 (12%)	28 (3.0%)
	4	21 (2.7%)	197 (25%)	213 (23%)
	5	120 (15%)	111 (14%)	210 (22%)
	6	147 (19%)	106 (13%)	252 (27%)
	7	105 (14%)	75 (9.4%)	211 (23%)
**Following Politics**				
	1	12 (1.5%)	40 (5.0%)	54 (5.8%)
	2	53 (6.8%)	79 (9.9%)	95 (10%)
	3	77 (9.9%)	85 (11%)	103 (11%)
	4	111 (14%)	145 (18%)	134 (14%)
	5	220 (28%)	194 (24%)	231 (25%)
	6	179 (23%)	166 (21%)	188 (20%)
	7	125 (16%)	87 (11%)	130 (14%)

Draft

Lastly, respondents were asked a series of questions related to the persuasion capacity of the article on a hypothetical reader with supportive, indifferent, or opposing attitudes towards climate change policies. The persuasion queries presented to participants in the first survey adhered to a specific format, such as: “Reader 1 believes that climate change is *not a problem* and is *opposing* national and international actions to combat climate change. After reading the article Reader 1 will:”. Participants were then given three response options to encapsulate the potential direction of opinion shift: becoming less favorable, remaining unchanged, or becoming more favorable towards actions intended to combat climate change. Similar questions were posed regarding hypothetical individuals who were indifferent or supportive of climate change policies.

To increase attention and compel respondents to think well about their answers, we also asked respondents to motivate

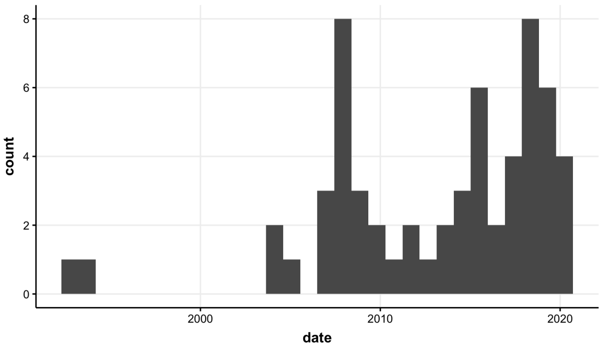

Figure S5. Distribution of publication years for the 60 articles utilized in the second survey task and the randomized experiment.

Table S2. Source of 1,350 articles used in the initial survey, along with their number of articles. Because of the long tail of the distribution, the table only shows the name of top publishers which had at least 10 articles within our sample.

**Source Name**	**Number of Articles**
US Fed News	22
Associated Press Newswires	21
Xinhua News Agency	21
Gene Therapy Weekly	19
Reuters News	19
Global Warming Focus	18
All Africa	17
The Guardian	17
The Canadian Press - Broadcast wire	16
Targeted News Service	12
The Times of India	12
The Herald	11
The Hindu	11
The Times	11
The Daily Telegraph	10

their choices about the climate stance of the article and its persuasiveness.

Notably, we intentionally withheld the source of these articles from the respondents, displaying only the title and publication date. This design decision aimed to eliminate the influence of source credibility, thus enabling a focus solely on content.

Respondent characteristics

For the task of article labeling, we recruited a total of 798 respondents via the Prolific platform. The sample comprised 402 individuals identifying as liberals and 396 as conservatives. After removing respondents who did not pass the comprehension or attention check questions[2] the final tally stood at 777 respondents, including 385 liberals and 392 conservatives. Table S1 presents the summary statistics of respondent characteristics. The demographic composition of the participants was designed to ensure a balanced representation across diverse political spectra, given the strong correlation between political affiliations and attitudes toward climate change, rather than to mirror the wider U.S. population. To accommodate a wide array of perspectives and curtail potential bias, each article was assessed by approximately four respondents, comprising an equal distribution of liberals and conservatives. This methodology aimed to account for the potential influence of the participants' political inclinations on their perception and interpretation of the articles. In the end, a total of 6,893 labels were collected for the articles.

[2] Overall, 21 respondents failed the comprehension check questions.

Average time and pay

The survey lasted on average about 47 minutes and paid a fixed reward of 6USD; accordingly the hourly wage was around 7.5USD/h.

Survey Step 3: Predicting persuasiveness

We conducted a second survey with the aim of collecting additional labels, enabling more precise estimates and ensuring full balance across various climate stance groups. In contrast to the first survey, which collected labels equally from liberals and conservatives, the second survey ensured balanced labels among skeptics, moderates, and advocates. This design choice was intended to align with the prediction question, where respondents forecasted the persuasive impact on three hypothetical readers with varying climate attitudes. Finally, we aimed to enhance the reliability and accuracy of article selection for the final experiment.

Article selection

To maximize relevance, we further constrained the set of 550 articles wherein the majority of labelers concurred that the main subject pertained to climate change. Towards this goal, we employed the following procedure.

The responses that participants gave in the first survey were encoded as (-1, 0, +1), and the average predicted persuasion score was computed at the article level by averaging these scores. Based on these average scores, the articles were ranked to identify the ones predicted to be the most persuasive for each stance group. Because the opinions of climate advocates could only experience reinforcement as opposed to persuasion, we focused only the predictions for skeptics and moderates, aiming to include 30 articles for each of these two groups. This design choice was made in order to maximize our chances of detecting a meaningful effect in the subsequent experiment. To counteract biases originating from labelers' stances, each set of 30 articles consisted of a mix of persuasive articles chosen by each stance group. Consequently, we tried to create a set of 60 articles that met the following criteria:

- 10 articles predicted to be most persuasive for individuals *opposing* climate policies, as determined by *Advocates*.
- 10 articles predicted to be most persuasive for individuals *indifferent* to climate policies, as determined by *Advocates*.
- 10 articles predicted to be most persuasive for individuals *opposing* climate policies, as determined by *Moderates*.
- 10 articles predicted to be most persuasive for individuals *indifferent* to climate policies, as determined by *Moderates*.
- 10 articles predicted to be most persuasive for individuals *opposing* climate policies, as determined by *Skeptics*.
- 10 articles predicted to be most persuasive for individuals *indifferent* to climate policies, as determined by *Skeptics*.

Draft

However, the sets of articles identified as persuasive by different stance groups were not mutually exclusive, leading to partial overlaps. As a result, the selection process for the most persuasive articles became more complex, requiring meticulous consideration of overlapping entries and a balanced composition within the sets. Two primary sources contributed to this overlap. Firstly, individual groups of labelers, such as advocates, might find the same articles persuasive for both moderates and skeptics. Secondly, the top persuasive articles selected for a particular target group could overlap with those identified for another group. For instance, both advocates and moderate labelers might find Article A persuasive for skeptics.

In the analysis of the latter scenario, the intersection of articles deemed persuasive by advocates for both moderates and skeptics included six articles. The intersections identified by moderate and skeptic labelers contained four and six articles, respectively. Regarding the former source of overlap, one intersection existed among the articles chosen by advocates and moderates for a skeptic audience, and one intersection among those selected by moderate and skeptic labelers. A similar pattern emerged for articles targeting a moderate audience. Notably, in both contexts, there was no intersection between the top articles that advocates and skeptics found persuasive.

To counteract these overlaps, we utilized a modified approach. Initially, the sets of articles chosen by each labeler stance for both categories of target individuals (those against or indifferent to climate policies) were combined. Subsequently, taking into account the persuasion rankings of the articles, we adjusted the ranking threshold to include a total of 20 unique articles. This approach was designed to ensure balanced representation across different labeler stances and foster diversity within the experiment's stimuli set.

Figure S5 shows the distribution of publication years for the 60 articles utilized in the second survey task and the experiment.

Thematic analysis

The sample of 60 articles deemed most persuasive encompassed a wide array of topics. To gain a deeper understanding of these topics and identify overarching themes, we conducted a thematic analysis using GPT-4o. In this analysis, we supplied GPT-4o with the text of the articles and tasked it with identifying common themes. Table S3 summarizes the list of themes provided by GPT-4o.

Structure

The main structure of the second survey remained largely consistent with that of the first survey, with the change of having only two training articles (one in favor and one against climate actions). In an effort to bolster survey credibility and mitigate participant fatigue, each respondent was assigned to review only three articles. The selection of articles provided balanced representation from the different stance groups, with one article selected by advocates, one by skeptics, and one by moderates. The sequence in which the articles were presented was randomized to counteract any potential order bias.

In this phase, respondents were asked to rate the articles' stance with respect to the severity of the climate changes issues. The histogram of these responses is shown in Figure S6, with answers coded on a 5-point scale: 1 representing 'No problem' and 5 representing 'Very serious problem.' The average response is 4.05, indicating that participants generally perceive the articles as portraying climate change as close to a 'Serious problem.'

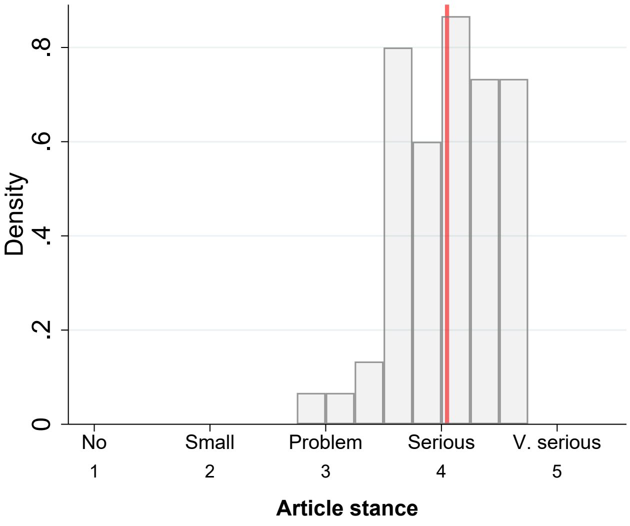

Figure S6. Distribution of the average score assigned to article in the question whether climate change is a problem (on a 5-point Likert scale). The vertical red line depicts the mean value in the distribution.

A significant modification was made to the final persuasion prediction question to obtain more nuanced data and bolster statistical power. Rather than providing discrete options, we introduced a continuous scale using a slider that ranged from -10 to 10 [1]. Accordingly, the question was framed as follows: "Reader 1 believes that climate change is *not a problem* and is *opposing* national and international actions to combat climate change. After reading the article, on a scale from -10 to +10, Reader 1 will become . . . ". Verbal descriptors indicating less favorable or more favorable positions were provided at the extremes, with the midpoint set at zero, to represent an unchanged stance.

Respondent characteristics

For the article labeling process in the second survey, a total of 794 respondents were recruited via the Prolific platform. One significant divergence from the first survey was the emphasis placed on achieving a nearly balanced distribution of respondents across different stance groups. This breakdown comprised 308 advocates, 259 skeptics, and 227 moderates. Given the lower prevalence of skeptics in the general population, we applied Prolific's filters for individuals with low climate stance.[3] In an effort to maintain balance at the article level, our aim was to gather approximately 10 labels from each stance group for each article. Figure S7 presents the distribution of labels assigned to articles across the different stance groups. As evident in the figure, slight variation exists in the number of labels allocated to different articles due to the iterative labeling process that was conducted in separate batches to

[3] Filters included the questions "Do you believe in climate change?" (selected answers for skeptics= No, Don't know, Not applicable / rather not say) and "Generally speaking, how concerned are you about environmental issues?" (selected answers for skeptics=1 (Not at all concerned), 2, 3; moderates= 2, 3; advocates=4, 5 (Very concerned)).

Draft

Table S3. Common themes identified in the 60 climate change articles deemed most persuasive, as generated by GPT-4o.

Theme	Description
Integration of Scientific Data	Articles frequently cite scientific studies, reports, and expert opinions to validate claims, such as the IPCC reports, studies from universities, and findings from organizations like WWF and IFAD.
Emphasis on Urgency	Many pieces stress the immediate need for action, often framing climate change as an urgent crisis that requires prompt attention and significant policy changes to mitigate future risks.
Local and Regional Impacts	Despite a global context, articles often highlight specific local or regional impacts of climate change, such as the effects on the Western Isles' ferry services, the heatwaves in Alaska, and biodiversity in Queensland.
Personal and Community Stories	Some articles incorporate personal stories or community-led initiatives to make the issue relatable, like Greta Thunberg's activism, local tree planting efforts, or testimonials from individuals affected by climate change.
Government and Policy Advocacy	Calls to action directed at governments and policymakers are common, urging them to adopt more stringent regulations, invest in clean energy, and lead by example, as seen in various appeals to national and international bodies.
Economic Integration	The economic impacts of climate change and the economic benefits of taking action are frequent discussion points. Articles mention both the costs of inaction and the potential for job creation and economic growth through green initiatives.
Public Awareness and Mobilization	Efforts to raise public awareness through marches, campaigns, and educational events are highlighted, indicating a push for grassroots mobilization to support broader climate initiatives.
Technological Solutions and Innovations	Many articles discuss existing and emerging technologies aimed at reducing carbon emissions and enhancing sustainability, such as renewable energy projects, green building initiatives, and advanced agricultural practices.
International Cooperation	The need for global cooperation and alignment on climate policies is a common theme, with references to international agreements like the Paris Agreement, COP meetings, and collaborative efforts between countries.
Multifaceted Consequences	The wide-ranging impacts of climate change are discussed, including health risks, environmental degradation, economic challenges, and societal disruptions, providing a comprehensive view of the issue.
Controversies and Challenges	Articles often touch on the controversies or challenges in implementing climate policies, such as opposition from fossil fuel industries, political disagreements, and the balance between economic development and environmental protection.
Success Stories and Positive Examples	Highlighting successful initiatives and positive examples, such as cities achieving emission reductions or community projects successfully mitigating local climate impacts, serves to inspire and provide proof of feasible solutions.

ensure adequate participation from skeptics and moderates. Despite these minor variations, nearly all articles received the minimum target of 10 labels per stance group. This balance in stance representation led to improved prediction accuracy compared to the results of the first survey.

Average time and pay

The survey lasted on average about 16 minutes and paid a fixed reward of 2USD; accordingly the hourly wage was around 7.5USD/h.

Randomized Experiment Step 4: Testing predicted persuasiveness

Structure

The experiment was divided into two main parts. In the initial part, we employed a similar approach to the previous survey to identify climate change skeptics. Participants were presented with a series of demographic questions, followed by a set of questions aimed at understanding their stance on climate change. In addition to the questions used in the previous surveys, we included one more question to measure

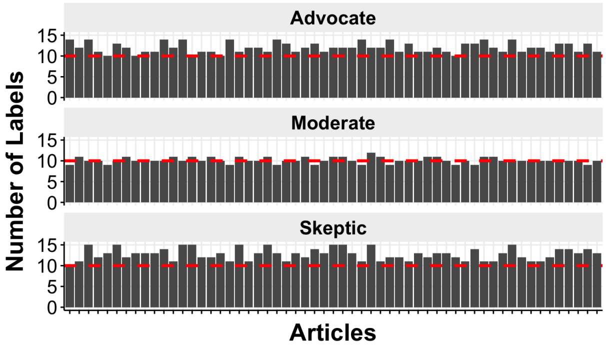

Figure S7. Label Count per Article in Second Survey, Categorized by Forecasters' Climate Stance. The second survey aimed to achieve a balanced distribution of label counts for each article, targeting approximately 10 labels from each stance group per article.

the extent of their belief in the urgency of climate change and the necessity of national and international actions to combat it. This question was carefully framed to mirror the same prediction question used in prior surveys, allowing us to capture their pre-treatment stance on the same construct accurately. After the initial phase, participants' stances were determined using the procedure described in Section **Research Design and Crowdsourced Tasks**. Those classified as climate change skeptics were given the option to participate in the second, previously unannounced, task for an additional bonus.

Outcome measurement

In the second part of the experiment, participants were asked to read one news article randomly chosen from either a set of 60 climate change articles or a control set of unrelated news articles. Approximately one-third of the participants were assigned to the control condition, while the remaining two-thirds received the treatment condition. After reading the article, participants were asked to respond to a set of questions aimed at eliciting their attitudes towards climate change. These questions covered three different dimensions: posterior beliefs in climate change, support for specific climate policies, and intention to engage in private actions that mitigate climate change. We adapted certain policy and action questions from the study conducted by Dechezleprêtre *et al.* [3]. Participants were instructed to rate their level of agreement with or support for each statement on a scale from 1 to 10. The statements included the following:

- **Belief**
  On a scale from 1 to 10, where 1 is "fully disagree" and 10 is "fully agree", how much do you agree with the following statements?
  - If nothing is done to limit climate change, there will be dire consequences for humanity in the not-distant future.
  - We urgently need national and international actions to combat climate change.
  - I am worried about climate change.
  - Human-caused climate change is real and it is occurring.
- **Policy Support**
  On a scale from 1 to 10, where 1 is "fully disagree" and 10 is "fully agree", what is your level of support for the following governmental policies to mitigate climate change?
  - Subsidize the insulation of buildings to make homes more energy efficient.
  - Subsidize the development and use of low-carbon technologies (e.g., renewable energy, capture and storage of carbon, etc.).
  - Impose a carbon tax on all products proportional to the amount of CO2 emitted for producing them.
  - Increase fuel duty, the tax motorists pay for petrol and diesel.
- **Personal Actions**
  On a scale from 1 to 10, where 1 is "I would never do it" and 10 is "I would certainly do it", what is your level of support for the following climate actions?
  - Increase walking, cycling, or using public transport instead of driving.
  - Use only green electricity, that is electricity produced by renewable energy, even if it costs more.
  - Vote for a candidate who is vocal about climate change issues.
  - Make a significant donation to an environmental cause.

We presented each set of questions related to posterior beliefs, support for climate policies, and intention to engage in private actions on separate pages to the participants. This allowed us to gather detailed and focused responses for each attitude dimension.

- **Donation to NGO for or against climate change**
  To complement the self-reported survey responses on participants' opinions and beliefs about climate change, we also incorporated a revealed preferences measure. Namely, we assessed participants' willingness to donate a share of their bonus received for participating in the survey, to one of four non-governmental organizations (NGOs). Among the four NGOs, two are in support of climate change mitigation ("Earth Justice" and "Sierra Foundation"), while the other two are against it ("Americans for Prosperity" and "Heartland"); the stance of these NGOs was made salient to the participants.

Respondent characteristics

The respondents were recruited from the Prolific online platform using filters for individuals with low climate stance (same procedure as in Step 3). Participants were initially screened to retain a sample of skeptics, based on their Climate Stance Index. A total of 1,687 respondents started the survey. Of these, 1,042 were classified as skeptics and were given the option to proceed to the second task for an extra bonus, with 1,001 choosing to continue. As stated in the pre-registration plan, we excluded any participant who met any of the following criteria: stated that they did not pay attention to the questions, performed straight-lining on at least two blocks of the main survey, read the article too quickly or spent less than two standard deviations below the mean time to complete the whole task, and provided nonsensical text in open-ended questions. Based on these constraints, 55 respondents were removed, resulting in a final sample of 946 skeptics. Furthermore, 11 observations were not considered in the analysis due to missing data on key outcome variables, resulting in a final sample of 935 observations.

Draft

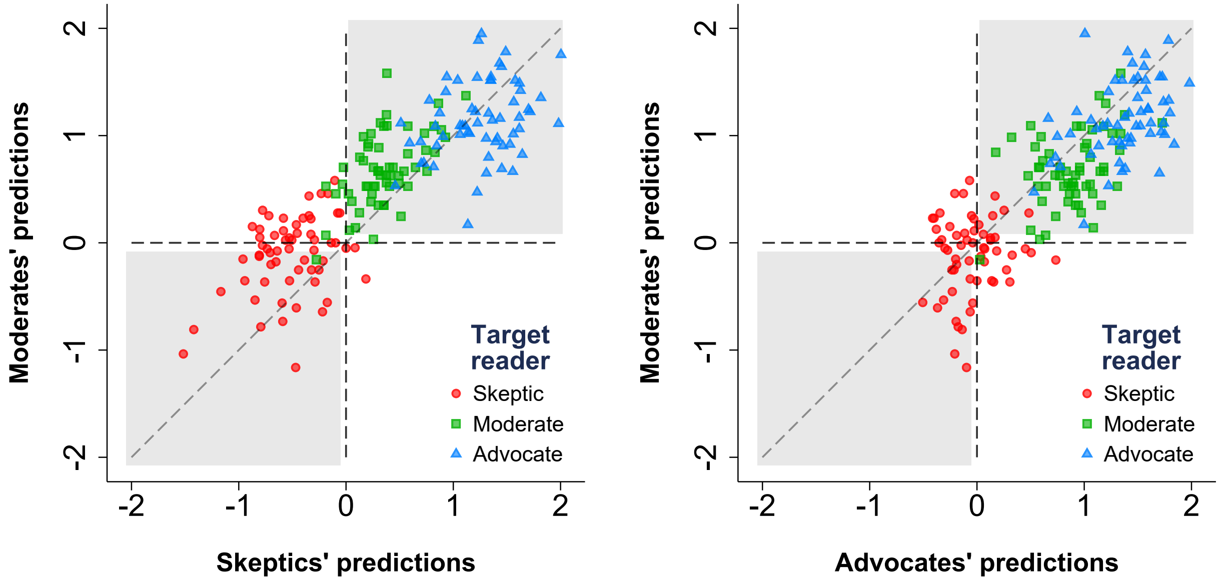

Figure S8. Predicted effects on climate change support at the article level. The axes depict the climate stance of skeptic and moderate (left) and advocate and moderate (right) forecasters. Each article is depicted three times in different colors, corresponding to different target reader groups for whom the predictions are being made(skeptics in green, moderates in red, and advocates in blue). The dashed diagonal line represents the identity line $y = x$, with articles closer to it indicating stronger agreement in the magnitude and direction of the predicted effect.

Draft

Average time and pay

The experiment lasted on average a bit more than 7minutes. The first part was shorter and had a fix reward of 0.3USD; the second (unannounced) part was longer and had an additional compensation of 1.7USD, a part of which could be donated to an NGO. Accordingly, the average hourly pay was slightly less than 17USD/h.

Average predictions by forecaster's stance

Figure S8 depicts the average predictions at the article level for different combinations of predictors stance. These results complement the result presented in Figure 2.

The Econometric Analysis

Aggregate treatment effects

We estimate the average treatment effect of being randomly assigned to the treatment group with the following regression model:

$$Y_i = \beta T_i + \delta b_i + \Gamma' \boldsymbol{C}_i + \epsilon_i \qquad (1)$$

The main independent variable is T_i, which is a binary treatment indicator that takes the value of 1 if the participant was assigned to the treatment group (exposed to the selected news articles about climate change) and 0 if the participant was in the control group (exposed to unrelated news articles). b_i represents the climate change belief question asked in the pre-treatment phase, to account for participants' baseline attitudes towards climate change policies. The vector $\boldsymbol{C}_i$ includes additional covariates, such as age, gender, education level, race, employment status, urbanity, social media activity, and political affiliations (democratic/republican scale and conservative/liberal scale). The inclusion of these covariates is not strictly necessary for model identification since the treatment assignment is randomized. However, including them can help improve the precision of the inference and account for any potential imbalances across the control and treatment groups. The error term ϵ_i represents the random variability or unexplained factors in the outcome.

The dependent variable, denoted as Y_i, represents the outcome of interest, which could be various measures related to respondents' attitudes towards climate change. To ensure reliable and robust measurements of respondents' attitudes, we created standardized indices for each outcome measure, including belief, policy, and actions. This was achieved by combining multiple items related to each attitude dimension into an index. By using this approach, we increased the stability of our survey measures, resulting in more precise estimates and greater efficiency in capturing true effects [1]. To standardize the indices, we standardized each component by subtracting the mean value in the control group, and dividing the result by the standard deviation in the control group. The indices were computed as averages over the equally weighted components[4]. This transformation allowed us to compare and interpret the results on a common scale, ensuring consistency across the different attitude dimensions. For the donation outcome, participants were asked to make a two-step decision. First, they selected one of four NGOs, two of which supported climate change mitigation and two that were against it. Next, they decided the amount they wanted to donate from their bonus. To capture this behavior, we encoded the donation amount as a continuous variable, taking negative values for donations to NGOs that act against climate change, 0 if no donation was made, and positive values for donations to NGOs that support climate change mitigation.

In the main text of the paper, we present the average treatment effects for the aggregate index, beliefs, policy support, individual actions, and donations. Figures S9 - S11 present estimated effects for the individual components of these

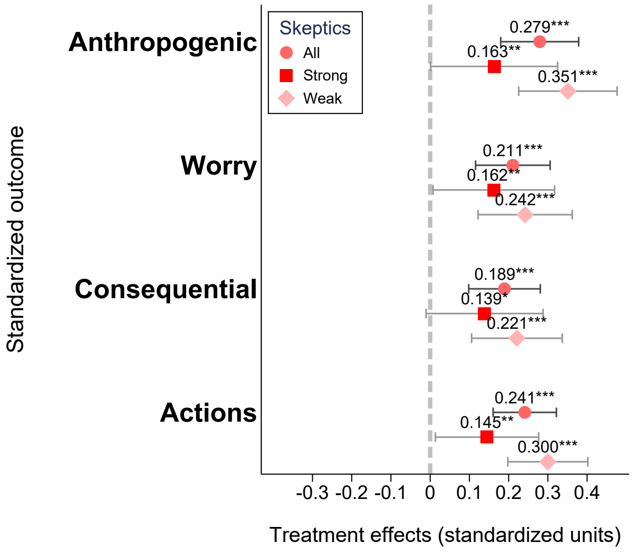

Figure S9. Average treatment effects on beliefs, by strength of skepticism. The outcome variables are standardized measures capturing four beliefs about the urgency of the climate change issue. For detailed information on outcome elicitation, please refer to section *Outcome measurement*. Following the method outlined by [4], each outcome variable is standardized by subtracting the mean of the control group and dividing by the control group's standard deviation. The estimated models include controls for respondents' pre-treatment climate change stance and socio-demographic characteristics. Respondents are categorized into two groups based on their pre-treatment climate change scores: Strong Skeptics (scores below 10) and Weak Skeptics (scores of 10 or higher). Error bars represent 95% confidence intervals around the estimated means. Statistical significance: *** p<0.01, ** p<0.05, and * p<0.1.

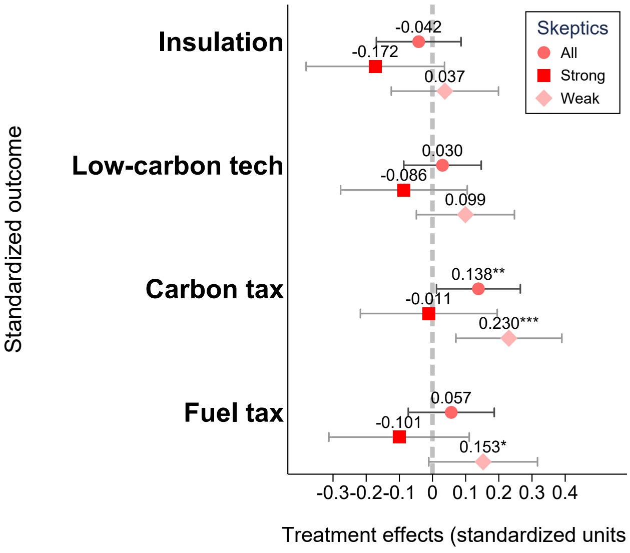

Figure S10. Average treatment effects on policy support, by strength of skepticism. The outcome variables are standardized measures that capture support for public policies addressing climate change. For detailed information on outcome elicitation, please refer to section *Outcome measurement*. Following the method outlined by [4], each outcome variable is standardized by subtracting the mean of the control group and dividing by the control group's standard deviation. The estimated models include controls for respondents' pre-treatment climate change stance and socio-demographic characteristics. Respondents are categorized into two groups based on their pre-treatment climate change scores: Strong Skeptics (scores below 10) and Weak Skeptics (scores of 10 or higher). Error bars represent 95% confidence intervals around the estimated means. Statistical significance: *** p<0.01, ** p<0.05, and * p<0.1.

Draft

measures. Figure S12 provides details on the distribution of donations in the treatment and control groups.

Alignment and misalignment in predictions

The Results section in the main text present the estimation results of average treatment effects when classifying climate change articles according to the alignment in predictions by skeptic and advocate forecasters. The results presented in Fig. 3A in the main text are derived from the estimation of the following regression model:

$$Y_i = \beta_1 TA_i + \beta_2 TM_i + \delta b_i + \Gamma' \boldsymbol{C}_i + \epsilon_i \qquad (2)$$

where the binary indicators TA_i and TM_i differentiate respondents assigned to articles with aligned and misaligned predictions, respectively. The corresponding coefficients, namely β_1 and β_2, measure the extent of actual persuasion for the two distinct subsets of articles. The remaining variables follow the definitions presented for the model in Equation 1. Table S4 presents the results of the estimation for our five outcomes, showcasing a separate estimation of treatment effects for the sets of articles classified as aligned, misaligned, and other.

Table S4. Estimated average treatment effects on main outcomes for the set of aligned and misaligned articles.

	Dependent variable				
	Index (1)	Belief (2)	Policy (3)	Actions (4)	Donations (5)
Aligned	0.080**	0.252***	0.062	0.012	-0.007
	(0.032)	(0.038)	(0.049)	(0.045)	(0.087)
Misaligned	0.068*	0.192***	0.027	0.063	-0.010
	(0.037)	(0.043)	(0.055)	(0.051)	(0.098)
Other	0.032	0.254***	0.010	0.183*	-0.319*
	(0.069)	(0.080)	(0.104)	(0.096)	(0.185)
Observations	935	935	935	935	935
Adj. R^2	0.582	0.766	0.402	0.404	0.014
Control mean	0	0	0	0	0
Wald test p-value	0.735	0.151	0.510	0.306	0.971
Notes:					*p<0.05; **p<0.01; ***p<0.001

Heterogeneous treatment effects by pre-treatment climate skepticism

In addition to estimating the average treatment effect, we explored how the impact of exposure to climate change articles varies depending on participants' baseline climate skepticism. To do this, we implemented a heterogeneous effects model by interacting the treatment indicator T_i with an indicator variable, S_i, representing whether a participant is a "strong" climate skeptic.

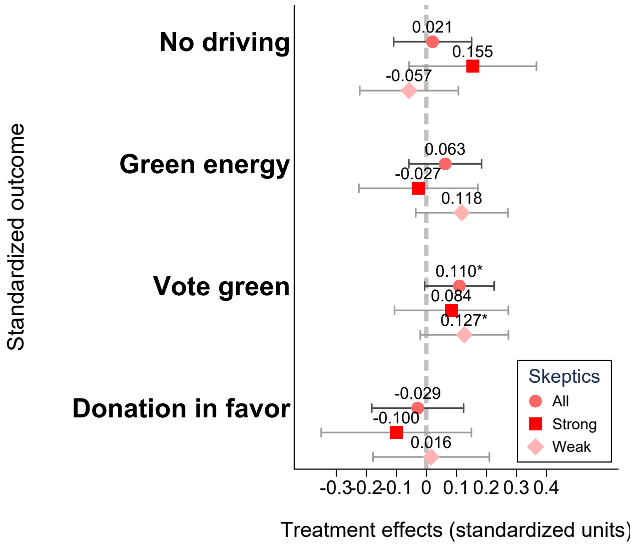

Figure S11. Average treatment effects on individual actions, by strength of skepticism. The outcome variables are standardized measures that capture intention to engage in individual actions addressing climate change. For detailed information on outcome elicitation, please refer to section *Outcome measurement*. Following the method outlined by [4], each outcome variable is standardized by subtracting the mean of the control group and dividing by the control group's standard deviation. The estimated models include controls for respondents' pre-treatment climate change stance and socio-demographic characteristics. Respondents are categorized into two groups based on their pre-treatment climate change scores: Strong Skeptics (scores below 10) and Weak Skeptics (scores of 10 or higher). Error bars represent 95% confidence intervals around the estimated means. Statistical significance: *** p<0.01, ** p<0.05, and * p<0.1.

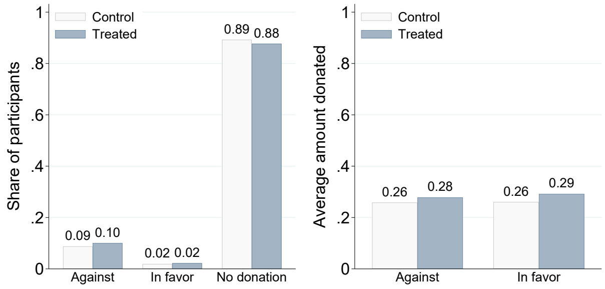

Figure S12. Share of donors and average donation amounts. The left panel displays the proportion of participants from both the treatment group (dark gray) and the control group (light gray) who chose to donate to an NGO either opposing climate change, supporting climate change, or opted not to donate at all. The right panel shows the average donation amounts from the treatment and control groups to an NGO either opposing or supporting climate change.

The results presented in Fig. 3B in the main text are derived from the estimation of the following regression model:

$$Y_i = \beta_1 T_i + \beta_2 (T_i \times S_i) + \beta_3 S_i + \delta b_i + \Gamma' \boldsymbol{C}_i + \epsilon_i$$

In this model, β_1 captures the baseline average treatment effect among weak climate skeptics (when $S_i = 0$). β_2 reflects the differential treatment effect for strong climate skeptics in comparison to weak skeptics. Thus, the total treatment effect for strong skeptics is given by $\beta_1 + \beta_2$. The term $T_i \times S_i$ represents the interaction between the treatment assignment and being a strong climate skeptic, allowing us to examine if the treatment's impact varies for this specific subgroup. Other variables remain consistent with the previous model: b_i accounts for baseline beliefs, and $\boldsymbol{C}_i$ controls for demographic and socioeconomic covariates to improve precision.

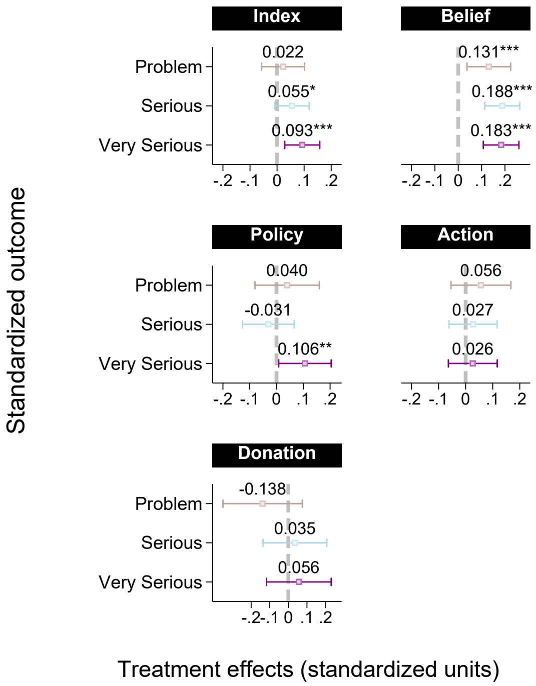

Figure S13. Treatment effects by severity of climate change. The model estimates the average effects of climate change articles, grouped based on the predominant response to the question: "According to the article, climate change is or could soon be:" with the following answer options: "No problem," "Small problem," "Problem," "Serious problem," and "Very serious problem." The 60 articles in our sample are classified by respondents in the prediction survey as belonging to one of the last three categories: "Problem," "Serious problem," or "Very serious problem." The estimation model controls for the standard set of covariates.

Additionally, we test the robustness of this specification by estimating an interaction model where the treatment indicator is interacted with a continuous measure of respondents' pre-treatment climate stance, rather than the binary indicator variable S_i. The estimated coefficients, presented in Table S5 of this appendix, reveal that the treatment is more effective in increasing the aggregate attitude index, beliefs, and policy support the less skeptical respondents are pre-treatment. Specifically, the interaction term is statistically significant for all three of these measures. However, for personal actions and donations, the treatment is neither effective nor does its effectiveness depend on pre-treatment climate concerns.

Table S5. Estimated average treatment effects on main outcomes in interaction with pre-treatment climate change belief.

	Dependent variable				
	Index	Belief	Policy	Actions	Donations
	(1)	(2)	(3)	(4)	(5)
Treated	-0.148	-0.042	-0.513***	0.189	-0.225
	(0.116)	(0.135)	(0.175)	(0.161)	(0.312)
Prior belief	0.072***	0.106***	0.055***	0.123***	0.006
	(0.010)	(0.012)	(0.016)	(0.014)	(0.028)
Treated × Prior belief	0.021**	0.027**	0.054***	-0.014	0.019
	(0.011)	(0.013)	(0.017)	(0.015)	(0.029)
Observations	935	935	935	935	935
Adj. R^2	0.584	0.767	0.409	0.403	0.013
Notes:			*p<0.05; **p<0.01; ***p<0.001		

Emotions

Figures S14 and S15 present the estimated treatment effects on the sets of positive and negative emotions, in support for the analysis presented in section **Results: Emotional Response** in the main text.

Our findings indicate that strong skeptics exhibit heightened feelings of hostility following the treatment compared to their counterparts in the control group. Similarly, weak skeptics exposed to the treatment report increased feelings of shame relative to weak skeptics in the control group. Additionally, we observe no other significant negative emotional responses and no significant changes in any of the five positive emotions.

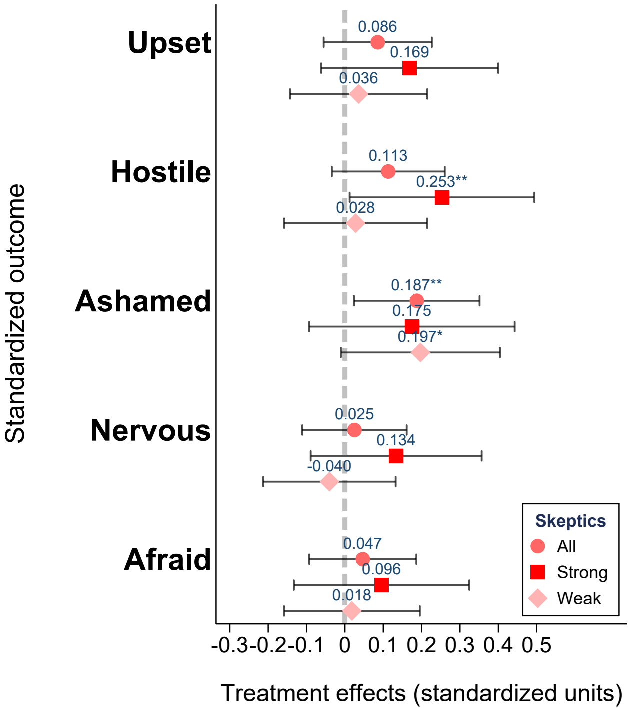

Figure S14. Average treatment effects on Skeptic readers' negative emotions. The outcome variables are standardized variables that capture five negative emotions, as measured by the International Positive and Negative Affect Schedule Short Form (I-PANAS-SF) scale [7]. The estimated models incorporate controls for respondents' socio-demographic characteristics. Respondents are categorized into two groups based on their pre-treatment climate change scores: "Strong Skeptics" (scores below 10) and "Weak Skeptics" (scores of 10 or higher). Error bars represent 95% confidence intervals around the estimated means. Statistical significance: *** p<0.01, ** p<0.05, and * p<0.1.

Predicted versus actual effects

This section supports the analysis presented in section **Predicted versus actual effects** of the main text. We compare the predicted versus actual effects, distinguishing the predictions by the forecaster's baseline climate stance.

Figure S18 illustrates the article-level average effects (aggregated index) along with their 95% confidence intervals depicted in gray. The x-axis displays the coefficients for each of the 60 articles arranged in ascending order based on the magnitude of actual effects. In color, the figure portrays the estimated average *predicted changes* for each article. In the left panel, the actual effects are compared to the predictions made by weak skeptics. In the right panel, the actual effects are compared to the predictions made by strong skeptics. Additionally, the figure presents the estimated coefficients of simple linear regression models, where actual effects are regressed on a constant and the predicted effects. The p-values for both coefficients are shown below the equation.

Our findings indicate that both weak and strong skeptics significantly underestimate the actual effects, as evidenced by the positive and highly significant intercepts in the linear regression models. However, the degree of underestimation is lower among weak Skeptics compared to strong skeptics. Additionally, both weak and strong skeptic forecasters appear to have limited ability to identify which articles will be more or less persuasive, as shown by beta coefficients close to zero and not statistically significant.

Robustness checks

To assess the robustness of our results to model specification, we replicated the main analyses of the randomized experiment using two additional models: one including random effects for articles and the other with standard clustering at the article level. The results are presented in Figures S16 and S17. As observed, while there are slight variations in p-values, the primary conclusions remain stable across these different model specifications.

Experimenter Demand Effect

The results of our experiment demonstrate that exposure to climate change information significantly increases expressed concerns about climate change among climate-skeptic readers. However, this heightened concern does not translate into increased support for climate policy, intended adoption of climate-friendly behaviors, or real-stake donations to or against climate change initiatives. We interpret these findings as reflecting a well-documented attitude-behavior gap, where individuals' stated preferences or concerns do not necessarily lead to corresponding actions. In this section, we discuss whether these results could instead be primarily driven by experimenter demand effects (EDE), where participants may adjust their self-reported attitudes in response to perceived expectations from the researcher.

Although experimenter demand effects are a valid concern in survey experiments, recent literature provides compelling evidence that challenges this critique [2, 8, 5]. Notably, [5] conduct a systematic review of EDE in survey experiments,

Draft

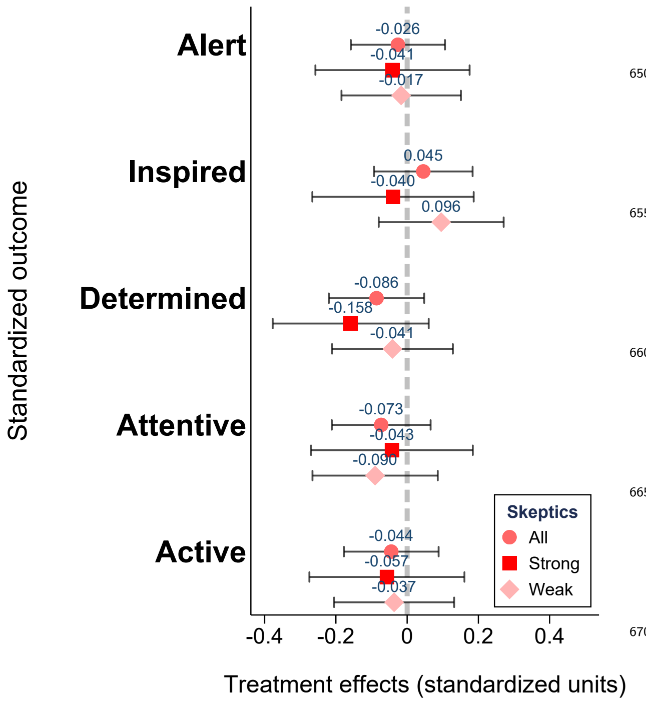

Figure S15. Average treatment effects on Skeptic readers' positive emotions. The outcome variables are standardized variables that capture five positive emotions, as measured by the International Positive and Negative Affect Schedule Short Form (I-PANAS-SF) scale [7]. The estimated models incorporate controls for respondents' socio-demographic characteristics. Respondents are categorized into two groups based on their pre-treatment climate change scores: "Strong Skeptics" (scores below 10) and "Weak Skeptics" (scores of 10 or higher). Error bars represent 95% confidence intervals around the estimated means. Statistical significance: *** p<0.01, ** p<0.05, and * p<0.1.

complemented by a series of rigorous tests, and conclude that such effects are generally minor and unlikely to meaningfully distort treatment effects.

In the following paragraphs, we provide arguments suggesting that EDE is likely minimal in our experiment, supported by the study design and observed patterns in the results. While it is impossible to entirely rule out the influence of EDE, we argue that our findings are more consistent with an attitude-behavior gap rather than demand-driven responses. Finally, in Section **Insights from open-ended feedback** the analysis of the open-ended feedback provided by survey respondents at the end of the task invigorate our argument.

- **Consistent Measurement across Control and Treatment Groups.** Our experimental design includes a control group that read an unrelated article, providing a baseline against which treatment effects are measured. All participants completed the same survey, with identical wording and structure for all outcomes, including concerns about climate change, policy support, intended behaviors, and real-stake donations. If participants inferred experimenter preferences, such perceptions would likely have influenced both groups equally, as the survey was uniform across treatments. These design features—comparison to a control group and consistent outcome elicitation—help mitigate concerns about experimenter demand effects.

- **Neutral Framing of Outcome Elicitation.** All questions used for outcome elicitation aimed to be framed as neutrally as possible, as detailed in Section *Outcome measurement* and as largely confirmed by the analysis of open-ended feedback (see Sec. **Insights from open-ended feedback**). For instance, to elicit concerns about climate change—the main variable where we estimate significant and strong treatment effects—participants were asked: "On a scale from 1 to 10, where 1 is 'fully disagree' and 10 is 'fully agree,' how much do you agree with the following statements?" This standard Likert scale minimizes directional cues and was designed to reduce the potential for bias in responses. Similarly, when eliciting real-stake donations, respondents were presented with a list of four NGOs—two advocating for climate change mitigation and two against it. These design features reduce the likelihood of experimenter demand effects by minimizing cues about the researchers' preferences.

- **Divergent Effects Across Stated Measures.** Our analysis focuses on four main outcomes: concerns about climate change, support for climate policies, intended climate actions, and real-stake donations. In the aggregate sample, only concerns are significantly affected by the treatment, while the other outcomes remain unchanged. This includes not only real-stake donations but also stated measures such as policy support and intended climate actions. This pattern is consistent with an attitude-behavior gap, where increased concerns do not translate into tangible actions or policy support. If experimenter demand effects were driving the results, we would expect all stated measures, not just concerns, to move similarly in response to the treatment.

- **Treatment Heterogeneity by Climate Stance.** Figure 3 (Panel B) in the main text highlights significant heterogeneity in treatment effects, with larger effects observed for weak skeptics compared to strong skeptics. This is particularly evident for climate concerns, where the effect size for weak skeptics is approximately twice as large as that for strong skeptics. Consistent with this, interaction models where the treatment dummy is interacted with a continuous variable capturing respondents' pre-treatment climate change attitudes reveal a statistically significant interaction term: individuals who were more concerned about climate change at baseline become even more concerned after treatment. These findings suggest that the treatment reinforces existing attitudes, leading to stronger effects among those already predisposed toward climate change concern (Table 5 in the supplemental material). Importantly, this pattern of heterogeneous effects minimizes concerns about experimenter demand effects, as such effects would likely result in uniform shifts across all respondents rather than being conditional on pre-treatment attitudes.

- **Treatment Effects and Climate Severity Framing.** Figure S13 provides suggestive evidence that treatment effects are slightly larger, particularly on the aggregate index, when the news articles describe climate change as a very serious issue compared to when it is framed as less of a serious problem. Although these differences are not statistically significant, the pattern suggests that the perceived severity of the issue amplifies the treatment effects. If strong experimenter demand effects were driving

Draft

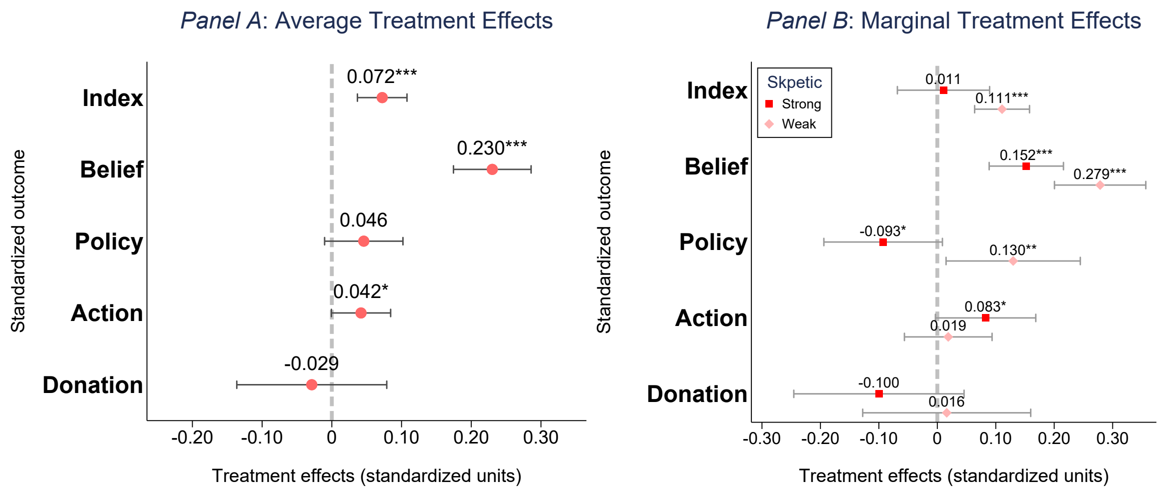

Figure S16. Average treatment effects on Skeptics' attitude towards climate change, standard error clustering at the article level. *Panel A* presents average treatment effects in the entire sample, *Panel B* presents marginal treatment effects, distinguishing among strong and weak Skeptic readers. The outcome variables are standardized indices capturing four climate support components: beliefs about the severity of climate change, support for climate policy, intention to adopt private mitigation actions, and actual donations to NGOs that are either supportive or against climate action. Additionally, the Index represents an average that equally weighs the four climate support components. The estimated models control for respondents' baseline climate change beliefs and socio-demographics; error bars represent 95% confidence intervals around the estimated means. In all models, standard errors are clustered at the article level (62 clusters). Statistical significance: *** p<0.01, ** p<0.05, and * p<0.1.

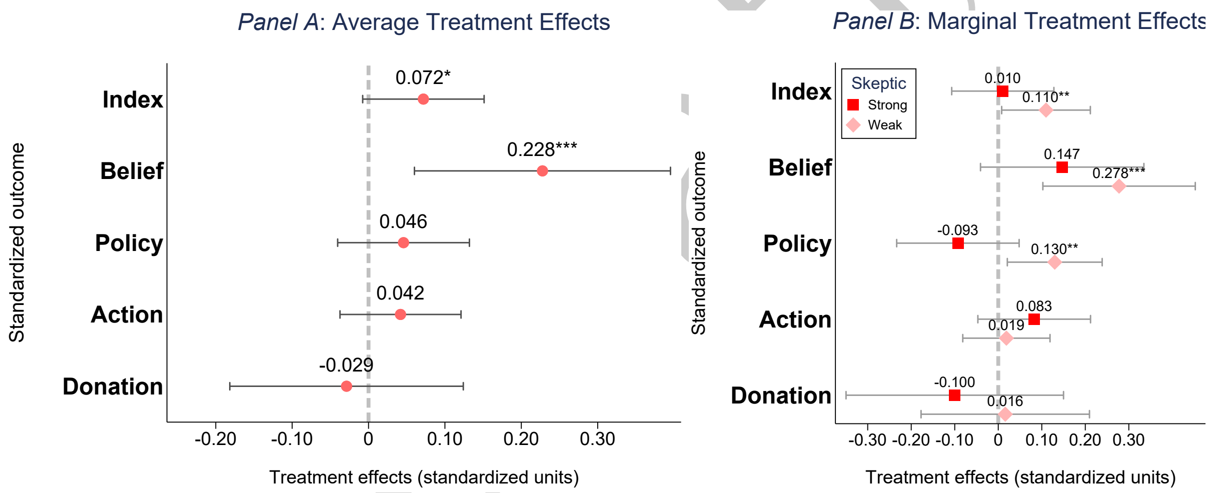

Figure S17. Average treatment effects on Skeptics' attitude towards climate change, random effects models. *Panel A* presents average treatment effects in the entire sample, *Panel B* presents marginal treatment effects, distinguishing among strong and weak Skeptic readers. The outcome variables are standardized indices capturing four climate support components: beliefs about the severity of climate change, support for climate policy, intention to adopt private mitigation actions, and actual donations to NGOs that are either supportive or against climate action. Additionally, the Index represents an average that equally weighs the four climate support components. The estimated models control for respondents' baseline climate change beliefs and socio-demographics; error bars represent 95% confidence intervals around the estimated means. The models are estimated with article-level random effects. Statistical significance: *** p<0.01, ** p<0.05, and * p<0.1.

the results, we would expect similar effects regardless of the framing, as participants would broadly align their responses with perceived researcher expectations. The observed variation in treatment effects based on article framing thus reduces concerns about EDE.

Insights from open-ended feedback

At the end of our experiment, respondents were asked to provide open-ended feedback of at least 50 characters about their experience in the task, guided by the following prompt:

Thank you for participating. We are very interested in your feedback about the following points:
-Was the task too long or too short?
-Did you feel you could express your opinion?
-Did you find any question unclear or uncomfortable?
-Did you feel that the survey was balanced, or rather biased towards the left or right?
-Did you experience any technical difficulty?
-How can we improve the study?

Our respondents wrote on average 209.3 (±11.6 CI) characters, with a minimum of 50 and max of 1,979. The length

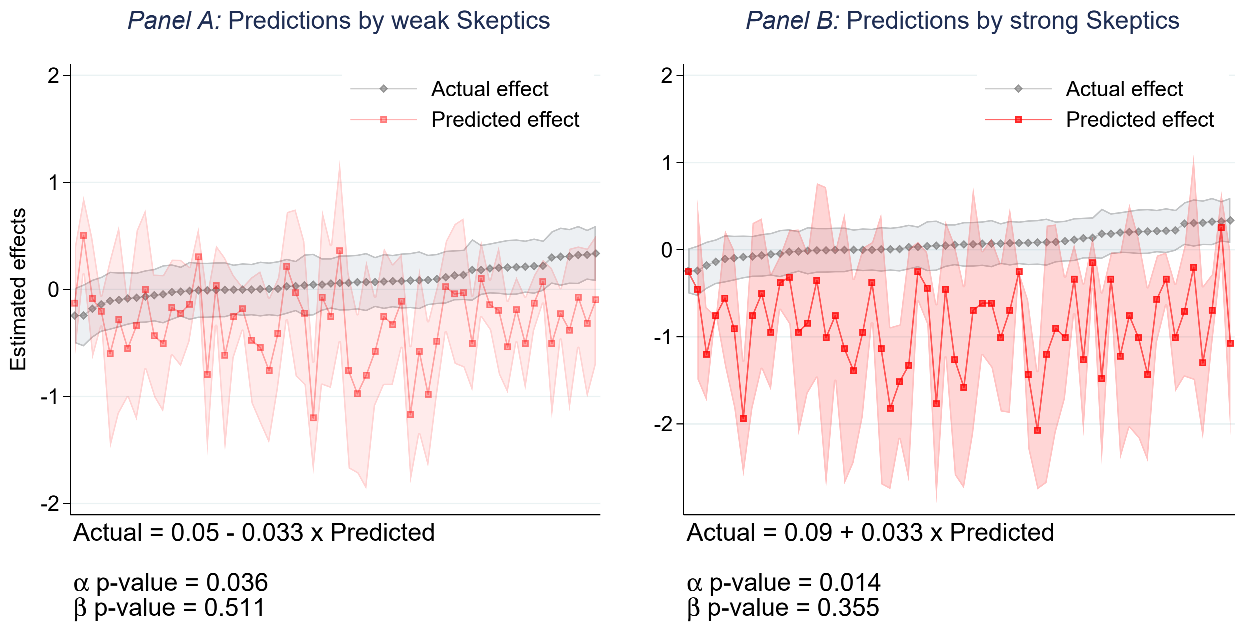

Figure S18. Predicted versus actual effects by weak and strong Skeptic forecasters. In each panel, the actual average effects and their 95% confidence intervals are depicted in gray. The average article-level predictions are depicted in green when forecasters are Skeptics (*Panel A*), in red when forecasters are Moderates (*Panel B*), and in blue when forecasters are Advocates (*Panel C*). The actual treatment effects for each article are estimated using OLS to fit the following model: $Y_i = \sum_{k=1}^{K} \beta_k I_k + X_i' \Gamma + \epsilon_i$, where Y_i is the index of climate support of the Skeptic reader i, $K = 60$ is the number of treatment articles, and I_k are dummy indicator for each treatment article. X_i is a vector of individual characteristics, and ϵ_i is the error term. To estimate the average predictions, we estimate the following model: $P_j = \sum_{k=1}^{K} \beta_k I_k + \epsilon_j$, where P_j is the prediction on the impact of article k by forecaster j. We distinguish between Skeptic, Moderate, and Advocate forecasters. Standard errors are clustered at the forecaster level.

Draft

of feedback is uncorrelated to treatment, political affiliation, and stance on climate change.

To analyze the open-ended text we used three different large language models (LLMs): Open AI GPT-4o, currently the state-of-the-art available commercially, the Google Gemini 1.5-flash-8b, a recent model of smaller size, and the open source model Llama 3.1 80 billion Instruct, which is particularly fit for tasks withcomplex instruction sets. Note that the first two models were accessed via API calls, while the latter one was configured and ran on a local GPU server at the University of Mannheim (equipped with two A100 NVidia chips).

We adopted standard prompt engineering practices and tasked all models to extract the respondents' answers to the six elicited criteria shown above and, in addition, to assign a value on a numerical scale to each of those. Requesting an explanation before a numerical answer is a common technique called "chain of thought" that enhances reliability and accuracy; finally, to further increase reliability, we set the temperature very close to zero in all models. The code used in this analysis and the exact prompt is available in the OSF project page.

Figure S19 shows substantial agreement across the three models across all six items, with some variation only in Panel D—whether participants found ways to improve the experiment. In relation to the experimenter demand effect, the following considerations can be made. First, all participants felt that they could express their opinion (Panel A; mean 6.2 ±0.1 CI on a 1-7 scale). Second, the task was clear and participants generally did not feel uncomfortable with it (Panel B; mean 6.5 ±0.1 on a 1-7 scale). Third, the majority of participants perceived the task as balanced with a slight bias pointing towards the Left (Panel C; mean 3.6 ±0.1 CI on a 1-7 scale). All in all, the findings in this section suggest that our results do not originate from an experimenter demand effect.

Sentiment of News Articles

We computed the most common words in the news articles of the experiment, after normalizing the corpus (removing stop words and HTML tags and lower-casing). The result are shown in Figure S20A. Not surprisingly, the top-4 words in the treatment articles are climate, change, global, warming. The next words are non-loaded terms such as report, emissions, energy, world, people. Overall, excluding risk and action, the remaining words appear to be either technical (e.g., emissions, levels) or terms related to nature (e.g., trees, sea).

To better quantify the sentiment of the articles, we performed a sentiment analysis using the dictionary-based package 'SentimentAnalysis' in R. This package automatically computes several scores using multiple dictionaries. Figure S20B shows the ranked distribution of sentiment for treatment articles using the dictionary QDAP (Quantitative Discourse Analysis Package), but similar results hold with other dictionaries. More than three quarters of the treatment articles lie in the "Neutral" category, that is with a score smaller than 0.1 and larger than -0.1. The remaining articles can be categorized as "Moderately positive."

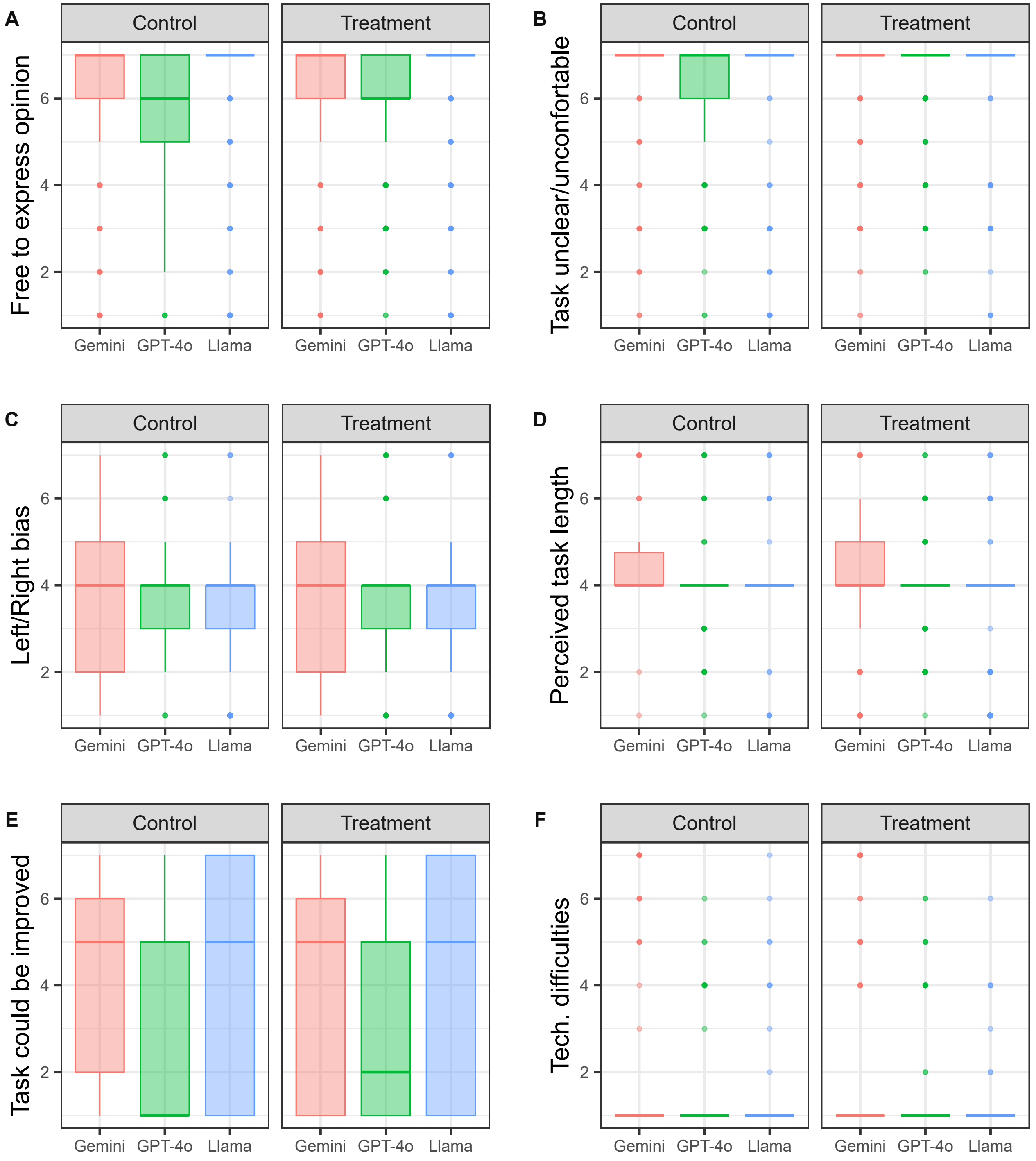

Figure S19. Open-ended feedback by survey respondents as analyzed by large language models. All models show the same qualitative results, namely: (i) participants could express themselves freely (Panel A), did not feel task was unclear or uncomfortable (B), perceived the task as balanced, but with a slight Left bias (C), the task was neither too long or too short (D, but GPT-4o model shows lower values), they had suggestions how to improve the task (E), and they did not experience technical difficulties (F). Gemini model is 1.5-flash-8b, LLama is 3.1 80 billion Instruct.

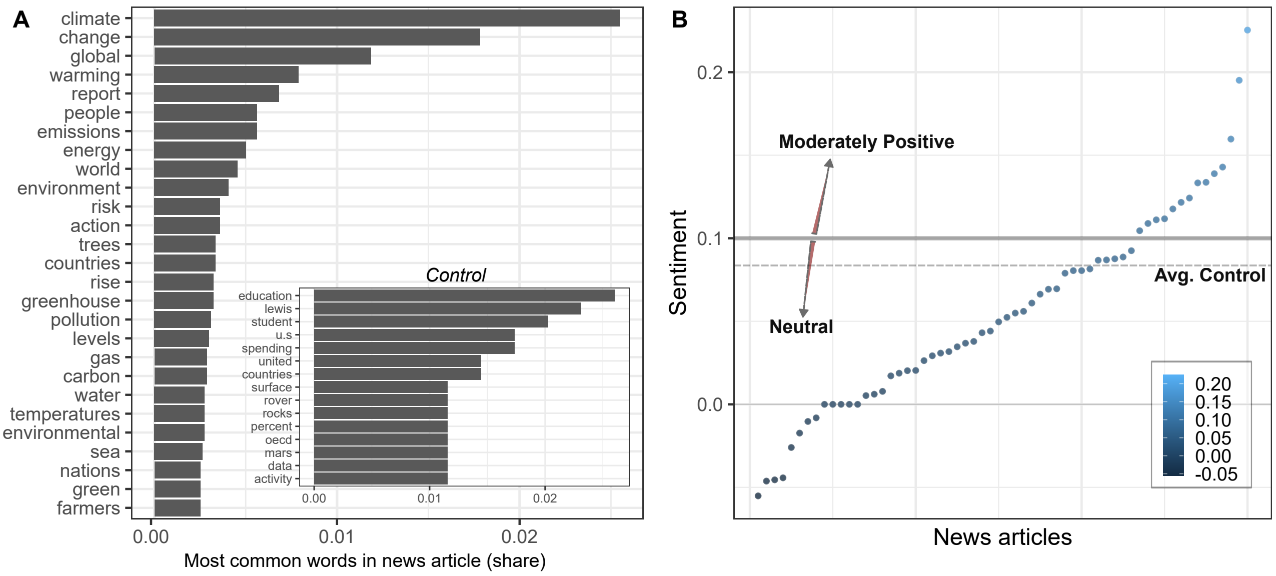

Figure S20. Most common words in the news articles of experiment and their sentiment. The share of most common words in the treatment and control (inset) articles (Panel A). The sentiment scores (QDAP) of the news articles is shown in Panel B. The majority of the treatment articles (46/60) is approximately neutral, and a bit less than one fourth moderately positive. The dashed line represents the average value for the two control articles. The larger gray line represents the limit between approximately neutral articles (less than 0.1) and those moderately positive (strongly positive articles would start from a score of 0.3). Both the words distribution and the sentiment scores suggest that the articles selected by the crowd were mostly neutral and not emotionally charged.

Draft

References

1. David E Broockman, Joshua L Kalla, and Jasjeet S Sekhon. The design of field experiments with survey outcomes: A framework for selecting more efficient, robust, and ethical designs. *Political Analysis*, 25(4):435–464, 2017.
2. Jonathan De Quidt, Johannes Haushofer, and Christopher Roth. Measuring and bounding experimenter demand. *American Economic Review*, 108(11):3266–3302, 2018.
3. Antoine Dechezleprêtre, Adrien Fabre, Tobias Kruse, Bluebery Planterose, Ana Sanchez Chico, and Stefanie Stantcheva. Fighting climate change: International attitudes toward climate policies. Technical report, National Bureau of Economic Research, 2022.
4. Jeffrey R Kling, Jeffrey B Liebman, and Lawrence F Katz. Experimental analysis of neighborhood effects. *Econometrica*, 75(1):83–119, 2007.
5. Jonathan Mummolo and Erik Peterson. Demand effects in survey experiments: An empirical assessment. *American Political Science Review*, 113(2):517–529, 2019.
6. Cass R Sunstein, Sebastian Bobadilla-Suarez, Stephanie C Lazzaro, and Tali Sharot. How people update beliefs about climate change: Good news and bad news. *Cornell L. Rev.*, 102:1431, 2016.
7. Edmund R Thompson. Development and validation of an internationally reliable short-form of the positive and negative affect schedule (panas). *Journal of cross-cultural psychology*, 38(2):227–242, 2007.
8. Ariel White, Anton Strezhnev, Christopher Lucas, Dominika Kruszewska, and Connor Huff. Investigator characteristics and respondent behavior in online surveys. *Journal of Experimental Political Science*, 5(1):56–67, 2018.

Draft
